# Different inflammatory signatures based on CSF biomarkers relate to preserved or diminished brain structure and cognition

**DOI:** 10.1038/s41380-023-02387-3

**Published:** 2024-01-12

**Authors:** Dayana Hayek, Gabriel Ziegler, Luca Kleineidam, Frederic Brosseron, Aditya Nemali, Niklas Vockert, Kishore A. Ravichandran, Matthew J. Betts, Oliver Peters, Luisa-Sophie Schneider, Xiao Wang, Josef Priller, Slawek Altenstein, Anja Schneider, Klaus Fliessbach, Jens Wiltfang, Claudia Bartels, Ayda Rostamzadeh, Wenzel Glanz, Katharina Buerger, Daniel Janowitz, Robert Perneczky, Boris-Stephan Rauchmann, Stefan Teipel, Ingo Kilimann, Christoph Laske, David Mengel, Matthis Synofzik, Matthias H. Munk, Annika Spottke, Nina Roy, Sandra Roeske, Elizabeth Kuhn, Alfredo Ramirez, Laura Dobisch, Matthias Schmid, Moritz Berger, Steffen Wolfsgruber, Renat Yakupov, Stefan Hetzer, Peter Dechent, Michael Ewers, Klaus Scheffler, Björn H. Schott, Stefanie Schreiber, Adelina Orellana, Itziar de Rojas, Marta Marquié, Mercè Boada, Oscar Sotolongo, Pablo García González, Raquel Puerta, Emrah Düzel, Frank Jessen, Michael Wagner, Augustín Ruiz, Michael T. Heneka, Anne Maass

**Affiliations:** 1 German Center for Neurodegenerative Diseases (DZNE), Leipziger Straße 44, Magdeburg, 39120 Germany; 2 Institute of Cognitive Neurology and Dementia Research (IKND), Otto-von-Guericke University, Magdeburg, Germany; 3 Department of Neurodegenerative Disease and Geriatric Psychiatry/Psychiatry, University of Bonn Medical Center, Venusberg-Campus 1, 53127 Bonn, Germany; 4 German Center for Neurodegenerative Diseases (DZNE), Bonn, Venusberg-Campus 1, 53127 Bonn, Germany; 5 Department of Neurodegenerative Disease and Geriatric Psychiatry/Neurology, University of Bonn Medical Center, Venusberg-Campus 1, 53127 Bonn, Germany; 6 Charité – Universitätsmedizin Berlin, corporate member of Freie Universität Berlin and Humboldt Universität zu Berlin, Institute of Psychiatry and Neuroscience, Hindenburgdamm 30, 12203 Berlin, Germany; 7 German Center for Neurodegenerative Diseases (DZNE), Berlin, Germany; 8 Department of Psychiatry and Psychotherapy, Charité, Charitéplatz 1, 10117 Berlin, Germany; 9 School of Medicine, Technical University of Munich; Department of Psychiatry and Psychotherapy, Munich, Germany; 10 University of Edinburgh and UK DRI, Edinburgh, UK; 11 German Center for Neurodegenerative Diseases (DZNE), Von-Siebold-Str. 3a, Göttingen, 37075 Germany; 12 Department of Psychiatry and Psychotherapy, University Medical Center Göttingen, University of Göttingen, Von-Siebold-Str. 5, 37075 Göttingen, Germany; 13 Neurosciences and Signaling Group, Institute of Biomedicine (iBiMED), Department of Medical Sciences, University of Aveiro, Aveiro, Portugal; 14 Department of Psychiatry, University of Cologne, Medical Faculty, Kerpener Strasse 62, 50924 Cologne, Germany; 15 German Center for Neurodegenerative Diseases (DZNE, Munich), Feodor-Lynen-Strasse 17, 81377 Munich, Germany; 16 Institute for Stroke and Dementia Research (ISD), University Hospital, LMU Munich, Feodor-Lynen-Strasse 17, 81377 Munich, Germany; 17 Department of Psychiatry and Psychotherapy, University Hospital, LMU Munich, Munich, Germany; 18 Munich Cluster for Systems Neurology (SyNergy) Munich, Munich, Germany; 19 Ageing Epidemiology Research Unit (AGE), School of Public Health, Imperial College London, London, UK; 20 Sheffield Institute for Translational Neuroscience (SITraN), University of Sheffield, Sheffield, UK; 21 Department of Neuroradiology, University Hospital LMU, Munich, Germany; 22 German Center for Neurodegenerative Diseases (DZNE), Rostock, Germany; 23 Department of Psychosomatic Medicine, Rostock University Medical Center, Gehlsheimer Str. 20, 18147 Rostock, Germany; 24 German Center for Neurodegenerative Diseases (DZNE), Tübingen, Germany; 25 Section for Dementia Research, Hertie Institute for Clinical Brain Research and Department of Psychiatry and Psychotherapy, University of Tübingen, Tübingen, Germany; 26 Division Translational Genomics of Neurodegenerative Diseases, Hertie Institute for Clinical Brain Research and Center of Neurology, University of Tübingen, Tübingen, Germany; 27 Department of Psychiatry and Psychotherapy, University of Tübingen, Tübingen, Germany; 28 Department of Neurology, University of Bonn, Venusberg-Campus 1, 53127 Bonn, Germany; 29 Excellence Cluster on Cellular Stress Responses in Aging-Associated Diseases (CECAD), University of Cologne, Joseph-Stelzmann-Strasse 26, 50931 Köln, Germany; 30 Division of Neurogenetics and Molecular Psychiatry, Department of Psychiatry and Psychotherapy, Faculty of Medicine and University Hospital Cologne, University of Cologne, Cologne, Germany; 31 Department of Psychiatry & Glenn Biggs Institute for Alzheimer’s and Neurodegenerative Diseases, San Antonio, TX USA; 32 Institute for Medical Biometry, University Hospital Bonn, Venusberg-Campus 1, D-53127 Bonn, Germany; 33 Berlin Center for Advanced Neuroimaging, Charité – Universitätsmedizin Berlin, Berlin, Germany; 34 MR-Research in Neurosciences, Department of Cognitive Neurology, Georg-August-University Goettingen, Goettingen, Germany; 35 Department for Biomedical Magnetic Resonance, University of Tübingen, 72076 Tübingen, Germany; 36 Leibniz Institute for Neurobiology, Brenneckestr. 6, 39118 Magdeburg, Germany; 37 Center for Behavioral Brain Sciences, Magdeburg, Germany; 38 Department of Neurology, Otto-von-Guericke University Magdeburg, Leipziger Strasse 44, 39120 Magdeburg, Germany; 39 Research Center and Memory Clinic. Ace Alzheimer Center Barcelona – Universitat Internacional de Catalunya, Barcelona, Spain; 40 CIBERNED, Network Center for Biomedical Research in Neurodegenerative Diseases, National Institute of Health Carlos III, Madrid, Spain; 41 Luxembourg Centre for Systems Biomedicine (LCSB), University of Luxembourg, 7 avenue des Hauts Fourneaux, 4362 Esch-sur- Alzette, Luxembourg; 42 Department of Infectious Diseases and Immunology, University of Massachusetts Medical School, 55 Lake Avenue, North Worcester, MA 01655 USA

**Keywords:** Neuroscience, Predictive markers

## Abstract

Neuroinflammation is a hallmark of Alzheimer’s disease (AD) and both positive and negative associations of individual inflammation-related markers with brain structure and cognitive function have been described. We aimed to identify inflammatory signatures of CSF immune-related markers that relate to changes of brain structure and cognition across the clinical spectrum ranging from normal aging to AD. A panel of 16 inflammatory markers, Aβ42/40 and p-tau181 were measured in CSF at baseline in the DZNE DELCODE cohort (*n* = 295); a longitudinal observational study focusing on at-risk stages of AD. Volumetric maps of gray and white matter (GM/WM; *n* = 261) and white matter hyperintensities (WMHs, *n* = 249) were derived from baseline MRIs. Cognitive decline (*n* = 204) and the rate of change in GM volume was measured in subjects with at least 3 visits (*n* = 175). A principal component analysis on the CSF markers revealed four inflammatory components (PCs). Of these, the first component PC1 (highly loading on sTyro3, sAXL, sTREM2, YKL-40, and C1q) was associated with older age and higher p-tau levels, but with less pathological Aβ when controlling for p-tau. PC2 (highly loading on CRP, IL-18, complement factor F/H and C4) was related to male gender, higher body mass index and greater vascular risk. PC1 levels, adjusted for AD markers, were related to higher GM and WM volumes, less WMHs, better baseline memory, and to slower atrophy rates in AD-related areas and less cognitive decline. In contrast, PC2 related to less GM and WM volumes and worse memory at baseline. Similar inflammatory signatures and associations were identified in the independent F.ACE cohort. Our data suggest that there are beneficial and detrimental signatures of inflammatory CSF biomarkers. While higher levels of TAM receptors (sTyro/sAXL) or sTREM2 might reflect a protective glia response to degeneration related to phagocytic clearance, other markers might rather reflect proinflammatory states that have detrimental impact on brain integrity.

## Introduction

The immune system plays a critical role in Alzheimer disease (AD) and other neurodegenerative disorders, and alterations in both central and peripheral immune responses have been related to brain structure, vascular pathology and cognitive function (for reviews see e.g., [1–3]). Microglia and astrocytes are the main resident glia that mediate neuroinflammation through production of cytokines, chemokines and other neuroinflammatory molecules in the brain. The early phase of AD is characterized by complex cellular interactions between glial cells, neurons and the vasculature, in which initially benign reactions become chronic, resulting in an irreversible dyshomeostasis of the brain [4]. Misfolded and aggregated proteins (e.g., Aβ) or tissue damage can trigger an innate immune response that initially aids phagocytic clearance and supports tissue repair and homeostasis [1]. However, if release of proinflammatory mediators is sustained, chronic inflammation might contribute to neurodegeneration and white matter (WM) injury [5].

Several inflammation-related molecules, including soluble receptors, cytokines, chemokines or markers of the complement system can be measured in cerebrospinal fluid (CSF) and in peripheral blood. Studies in aging and AD have reported both positive and negative associations of individual inflammation-related markers with brain structure and with cognitive function, varying by the type of marker and disease stage.

Specifically, for some immune-related markers such as sTREM2, sTyro/sAXL positive relationships of CSF levels with gray matter (GM) volume and memory performance have been observed. For instance, higher sTREM2 levels in CSF were related to higher GM volume in temporal and parietal regions in patients with mild cognitive impairment (MCI) [6] and to attenuated cognitive decline in the A + T+ individuals when adjusting for AD biomarker levels [7]. Similarly, we recently observed that higher CSF levels of soluble TAM receptors sTyro and sAXL were related to higher GM thickness or volume in a priori regions of interest, to better cognition and less cognitive decline [8] in a sample of cognitively unimpaired (CU) and cognitively impaired (CI) individuals. CSF levels of YKL-40 have been related to higher or reduced brain volume depending on the AD-related disease stage [9]. Notably, in several studies the aforementioned inflammatory markers were increased in the presence of tau tangle pathology, even in non-demented individuals [7, 8, 10] and positive relationships with brain structure and cognition were most pronounced when adjusting for AD biomarkers [7, 8]. Taken together, this indicates that these CSF markers are increased as part of a damage response and may provide access to mechanisms of brain protection. Alternatively, the observed cross-sectional positive associations with brain volume and cognition could be due to brain swelling or to brain reserve, i.e., subjects with higher brain volume can bear higher levels of these inflammatory markers while remaining cognitively normal. Ultimately, longitudinal MRI data is needed to support the hypothesis of neuroprotective effects by showing reduced atrophy over time (see e.g., [7]). Whether the “beneficial” effects on brain structure are also observed with respect to WM integrity is an open question. As a proxy, white matter hyperintensities (WMHs) are thought to primarily reflect microvascular lesions and thus represent a marker of (subclinical) cerebrovascular disease [11] and WMHs have been associated with inflammation and reduced protein clearance (for review see, [12]).

In contrast, proinflammatory markers such as interleukin (IL)-6 and C-reactive protein (CRP) measured in blood have been related to reduced brain integrity and worse cognition in large samples of older adults [13–15] and in middle-aged adults [16]. Although relationships with WM lesions (e.g., [17]) or atrophy over time [13] have not been always observed, studies on peripheral marker levels point overall towards detrimental effects of CRP and certain proinflammatory ILs on brain integrity. Peripheral inflammatory mediators can cross the blood-brain barrier to modulate central inflammatory processes. Studies that measured these markers directly in CSF are rare and their associations with brain structure and memory function need to be further explored [18].

In summary, previous findings suggest that certain inflammatory markers relate to increased or decreased brain integrity and cognitive function, which could represent different inflammatory processes and also depend on the clinical-pathological disease stage. This raises the question if different “inflammatory signatures” (indicative for shared biological processes) can be derived from inflammatory CSF biomarkers by multivariate analysis that are related with either preserved or degraded brain integrity and cognition. Most previous studies only measured a single or a few inflammatory markers, and analyzed their association with brain structure or cognition individually. Moreover, most studies used cross-sectional neuroimaging data. This precludes a comprehensive picture on how groups of markers might be altered in parallel and show concordant associations with atrophy or cognitive decline.

In the current study, we aimed to address these open questions by analyzing a large, comprehensive panel of CSF immune-/inflammatory markers in the DELCODE cohort [19]. DELCODE is an observational longitudinal multi-center neuroimaging study in a sample of older adults that comprises individuals across the full risk spectrum of AD, including healthy controls (HCs), cognitively normal first-degree relatives of AD patients (ADRs), as well as patients with subjective cognitive decline (SCD), MCI, or mild dementia of the Alzheimer’s type (DAT). Specifically we aimed to address the following questions: 1) Are there groups of inflammatory markers (inflammatory signatures) that are beneficial or detrimental in terms of their relationship to brain structural integrity and cognition; 2) How are these groups of markers related to global and local structure including WM integrity/damage and 3) to subsequent changes in GM volume over time (i.e., is there evidence for protective effects); 4) Are relationships of inflammatory signatures with brain structure and cognition moderated by AD-related disease stage. Ultimately, main analyses were replicated within the framework of the EU-JPND funded PREADAPT project in a cohort of SCD and MCI subjects of the Fundacío ACE (F.ACE) Alzheimer Center, Barcelona [20].

## Methods

### Ethics approval and consent to participate

The general study protocol for DELCODE was approved by the ethics committees of the medical faculties of all participating sites. The process was led and coordinated by the ethics committee of the medical faculty of the University of Bonn (registration number 171/13). DELCODE was registered with the German Clinical Trials Register (DKRS study ID: DRKS00007966). Use of data and biomaterial for the specific work described in this manuscript was furthermore approved by the ethics committee of the Medical Faculty of the University of Bonn, reference No. 122/18.

### Study design

**Fig. 1:**
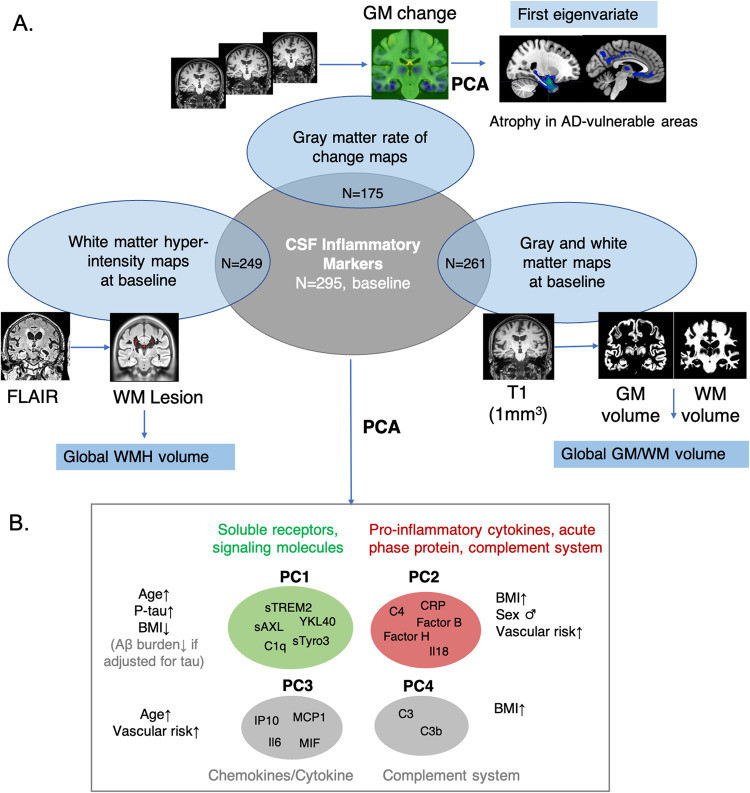
Overview of methods and derived summary inflammation and brain measures. **A** A panel of 16 immune-related markers was measured in CSF at baseline in the DELCODE sample. FLAIR and T1-weighted MR images at baseline were acquired in a subsample of participants. White matter hyperintensity (WMH) lesion maps were derived by segmentation of FLAIR images (LST toolbox). Gray matter (GM) and white matter (WM) volume probability maps were derived by segmentation of T1 images (CAT/SPM toolbox). As global summary measures we derived global WMH volume as well as GM and WM volume. GM rate of change maps were derived for subjects with 3 longitudinal T1-images using Jacobian integration (SPM). A PCA on the GM rate of volume change maps was performed within an AD vulnerable atrophy mask (see methods) to create subject-level summary measure (first eigenvariate) of atrophy over follow-ups. **B** A PCA on the immune markers resulted in 4 principal components (PCs). For each PC, the individual markers with highest loadings are depicted, which were all positive. See Supplementary Fig. 2 for the loadings.

The DELCODE cohort is a German multicenter observational study with details provided in [19]. Our study included a total of 295 subjects from the DELCODE cohort with available CSF inflammatory and AD markers (i.e., complete panel of all markers which are described below). The sample included 70 HCs, 22 ADRs, 97 SCDs, 69 patients with MCI and 37 with DAT. Of those, 261 subjects had baseline GM/WM volume maps, 249 had WMH data, 175 had longitudinal GM atrophy data and 204 longitudinal cognitive data (see Fig. 1 and Supplementary Fig. 1).

Normal cognition was defined as having memory test performances within 1.5 SD of the age-, gender-, and education- adjusted normal performance on all subtests of the CERAD (Consortium to Establish a Registry of AD test battery). SCD was defined as the presence of subjective cognitive decline as expressed to the physician of the memory center [21] and normal cognition as assessed with the CERAD. Participants were classified as MCI when displaying an age-, gender-, and education-adjusted performance below –1.5 SD on the delayed recall trial of the CERAD word-list episodic memory tests. The AD group consisted of participants with a clinical diagnosis of mild AD [22] obtaining ≥ 18 points on the Mini Mental State Examination (MMSE). All participants were 60 years or older, fluent speakers of German and had a relative or close friend who completed informant questionnaires.

### Cerebrospinal fluid (CSF) measures

Procedures of CSF acquisition, processing, and analysis in the DELCODE cohort have been previously described [19]. CSF measures of AD pathology included Aβ42/40 and phospho-tau181 (p-tau) as measures of Aβ (A) and tau pathology (T) were determined using commercially available manual colorimetric and electrochemiluminescence ELISA (by Fujirebio and Meso Scale Diagnostics, see [19]. For supplementary group analyses, we categorized individuals according to the AT(N) biomarker classification system [23]. Cut-off values for A/T biomarkers were based on Gaussian mixture modeling of the DELCODE data independent of any group assignments using the R package flexmix (version 2.3-15) [24] using the full CSF baseline sample including 527 individuals (T+: p-tau >= 73.65 pg/ml; A+: Aβ42/40 ≤ 0.08).

We investigated a panel of 16 CSF biomarkers with a focus on immune-/inflammatory markers related to different mechanisms of inflammation, immune regulation, and signaling including soluble receptors (sTREM2, sAXL, sTyro3), inflammatory signaling molecules (YKL-40, CRP, cytokines: Il-6, Il-18, chemokines: MCP-1, IP-10, MIF) and complement factors (C1q, C3, C3b, C4, B, H). The panel was determined by enzyme-linked immunosorbent assays (ELISA) and has been chosen because these markers are robustly detectable and previously showed associations with aging and AD features [8, 10, 25, 26]. Details on assay specifications are provided in [8].

### Voxel-based morphometry (VBM)

MRI image acquisition is described in the Supplementary (one image was excluded that did not pass quality assessment). The MPRAGE images were processed using SPM (SPM12 v7771, Statistical Parametric Mapping software; Wellcome Trust Centre for Human Neuroimaging, London, UK) and CAT-Toolbox (r1888, Structural Brain Mapping group, Jena University Hospital, Jena, Germany) running on MATLAB (r2016b, The MathWorks, Inc., Natick, Massachusetts, USA). The CAT approach performs a correction for field inhomogeneities and then segments images into GM, WM, and CSF including a partial volume estimation correction. These maps were also used to derive measures of whole brain GM and WM volume. The obtained tissue probability maps were then warped to a study-specific template in MNI space using SPM’s Geodesic Shooting approach. The tissue maps were modulated by the Jacobian determinant of the deformation fields to enable voxel-based comparisons of local GM volume across subjects. A Gaussian blurring kernel was applied using 6 mm full width half maximum (FWHM). The resulting tissue maps were quality checked using CAT’s sample homogeneity check, which revealed one extreme outlier for GM and one for WM probability maps in our sample, which were excluded from the analyses (final *N* = 258).

### Longitudinal brain morphometry

Individual rates of change of brain volume (atrophy) were first estimated in all 517 subjects of the DELCODE cohort who had complete availability of MPRAGE images at baseline and two annual follow-up measurements (mean time interval 376.7 ± SD 31.6). Individual diffeomorphic deformations were obtained using SPM’s longitudinal registration [27] incorporating bias correction, linear and nonlinear registration of all 3 timepoint scans. GM segmentations of each subject’s midpoint images were modulated and normalized to a study-specific template space. Slope images characterizing local GM volume changes were obtained using voxel-based linear models after 6 mm Gaussian smoothing. To derive a summary measure of AD-related GM change (atrophy), we first localized GM areas showing any clinical group differences (HC/ADR, SCD, MCI, DAT) at baseline (*p* < 0.05 FWE) in the full longitudinal sample of 517 subjects. Second, we used this as an AD-vulnerable mask and generated the subject-level rate of change in this mask via Principal Component Analysis using MATLAB’s eig function. The resulting first eigenvariate (see Fig. 1A) weighs voxel-level rates of GM change according to their variance in the sample and reflects individual differences of GM change. In addition, we also quantified mean GM volume change in the whole GM as a global measure of atrophy that is not specifically sensitive to AD-related atrophy. Finally, the GM slope images were used to assess voxel-wise associations between inflammation markers and change in GM (see statistical analysis). Longitudinal GM analyses were performed in 174 subjects with full CSF data and passed quality assessment (Supplementary Fig. 1).

### White matter hyperintensity (WMH) measures

WMH lesion segmentation and subsequent voxel-based lesion analysis was used to assess inflammation related differences of local lesion probability due to WMH. The pipeline involved lesion segmentation based on the Lesion Segmentation Toolbox ([28], https://www.applied-statistics.de/lst.html, v3.0.0). Lesion probability maps were derived using the Lesion Prediction Algorithm (LPA) which is recommended for use in multi-site studies [29, 30]. The LPA uses a logistic regression model of binary lesion data of multiple sclerosis patients with severe lesion patterns. The parameters of this model fit are then used to segment lesions in FLAIR baseline scans of the DELCODE cohort by providing an estimate for the WMH lesion probability for each voxel. Total lesion volume (after log transformation) was used as a quantitative proxy for vascular disease severity throughout the whole brain. WMH data were available for 249 subjects.

### Vascular risk score

We created a vascular risk-score based on the medical history of the DELCODE participants, which was coarsely based on the Framingham cardiovascular risk profile (FCRP) score that represents a predictive value for coronary heart disease [31]. Of the available pre-existing medical conditions, we selected three conditions to create a vascular risk-score by summing up indicators for hypertension, diabetes and abnormal fat metabolism to a score with values 0 to 3.

### Cognitive measures

For cognition at baseline, we used factor scores from a previous study for the following cognitive domains: episodic memory, language ability, executive functions, working memory and visuo-spatial abilities based on a confirmatory factor analysis on the extensive DELCODE neuropsychological battery [32]. For baseline analyses with inflammatory components, we focused on the episodic memory factor. Exploratory supplementary analyses further assessed associations between inflammatory markers and the other cognitive domains.

For assessment of longitudinal changes in cognitive performance, we used a preclinical Alzheimer´s cognitive composite (PACC5) [33]. The PACC5 is a neuropsychological composite measure that was designed to index cognitive changes in the early phase of AD. To construct the PACC5, we z-standardized and averaged the following tests: Free cued and selective reminding test (total and free recall), symbol digit modalities test, logical memory delayed recall, semantic fluency (animals) and the MMSE.

### Statistical analysis

#### PCA on inflammatory markers

Inflammatory data (log-transformed) was analyzed using SPSS 24 (IBM, Armonk, NY). Principal component analysis (PCA) enables to estimate components that represent shared variation in the markers, potentially indicative for shared biological processes. A single marker is prone to measurement error and might (in combination with others) index more than one process (or cell-type) i.e. markers showing substantial correlations. Therefore, a PCA, well established multivariate approach was applied to account for relationships between markers and reveal (more reliable) composite scores. The PCA was performed on the Z-standardized inflammatory biomarker data (default parameters: Kaiser’s criterion= eigenvalue > 1) in SPSS. Kaiser’s normalization was used where unrotated loadings are divided by the square roots of the communalities of the corresponding observed variables. We used an orthogonal rotation (Method: Varimax) to create uncorrelated components with ‘simple structure’ factor loadings in the rotated component matrix that are close to 1, −1 or 0.”

#### Analyses on brain summary measures and cognition

First, we performed descriptive correlational analyses to assess how the PCs were related to AD biomarkers and other demographics (reported p-values are uncorrected). As our previous study suggested that some inflammatory markers relate to less Aβ burden when accounting for tau burden [8], we also ran exploratory correlations between PCs and Aβ42/40 while covarying for p-tau. Associations between inflammatory PCs and global brain structural measures as well as memory were assessed by GLMs in SPSS. We ran individual regression models for each brain or cognitive measure including all four PCs as predictors and included age, gender, Aβ42/40 and p-tau as covariates of no interest in the model. For models on volumetric measures, we also included total intracranial volume (ICV) as a covariate. We also tested whether associations remained significant when accounting for BMI, APOE 4 status or vascular risk score. Additional sensitivity analyses were performed to determine how results are influenced when including CSF volume, global inflammatory marker levels or Aβ40 levels as covariates into the model as attempt to account for inter-individual differences in mean CSF protein levels [34]. And finally we tested how results change if diagnostic group is added to the models. These analyses are reported in the Supplementary.

We then assessed whether associations between PCs and brain/cognitive measures were moderated (differed) by the clinical-pathological stage across the AD spectrum (A-T- CU, A + CU, A + CI) by testing for an interaction between the components and stage (see Supplementary results).

The relationship between inflammatory components and cognitive change was analyzed by linear-mixed effects (LME) using the lme4 function in R (version 1.1–27.1) in subjects with availability of at least 3 visits (*N* = 204, *N* observations = 856). The LME included correlated intercepts and slopes as random effects. The four PCs, age, gender, Aβ42/40 and p-tau as well as their corresponding interactions with time were included as fixed effects.

#### Voxel-wise whole-brain analyses

VBM analyses were conducted to examine the patterns of local morphological relationships between inflammatory components (that showed significant associations with brain summary scores) with baseline differences of GM, WM and WMH volumes and GM volume slope images by performing individual multiple regressions in SPM12. Age, gender, Aβ42/40, p-tau and ICV were included as covariates.

### Effect replication in the F.ACE cohort

To test if the major findings made in DELCODE could be replicated in an independent cohort, we utilized samples and data of the F.ACE cohort, including subjects diagnosed with SCD and MCI (*n* = 185). The sample and analyses are described in detail in the Supplementary Material.

## Results

### Cohort demographics

Table 1 summarizes the demographic characteristics of the analyzed cohort. In brief, the sample analyzed here comprised 295 older individuals (48.5% females) with mean age of 71 years (SD = 6), 14 years of education (SD = 3), mean BMI of 26 (SD = 3) and prevalence of APOE 4 genotype of 37%. Of those, 189 were CU (HC, ADR, SCD; 64%) and 106 CI (MCI, DAT; 36%). AT-biomarker staging based on CSF levels revealed 163 A-T- (55%), 64 A + T- (22%), 7 A-T+ (2%) and 61 A + T+ (21%).

**Table 1:** Sample characteristics in DELCODE.

Feature	All	Cognitively unimpaired (CU)	Cognitively impaired (CI)
*N*	295	189	106
Diagnostic groups (N)		HC (70), ADR (22), SCD (97)	MCI (69), DAT (37)
Age (yrs.)	70.6 ± 5.8	69.5 ± 5.5	72.7 ± 5.9
*N* female (%)	143 (48.5)	98 (51.9)	45(42.5)
Yrs education	14 ± 3	14.7 ± 2.9	14.0 ± 2.9
BMI	26 ± 3	25.6 ± 3.2	25.6 ± 3.8
*N* APOE ε4 + (%)	108 out of 291(36.6)	55 out of 186 (29.1)	53 out of 105 (50.0)
Aβ42/40	0.083 ± 0.029	0.093 ± 0.024	0.064 ± 0.028
p-tau181 (pg/ml)	59.9 ± 30.3	50.9 ± 20.6	76.0 ± 37.4
*N* A-T- (%)	163 (55)	133 (70)	30 (28)
*N* A + T- (%)	64 (22)	40 (21)	24 (23)
*N* A-T+ (%)	7 (2)	5 (3)	2 (2)
*N* A + T+ (%)	61 (21)	11 (6)	50 (47)
Memory factor	−0.124 ± 0.988	0.436 ± 0.505	−1.122 ± 0.841

Unless otherwise stated variables denote mean ± standard deviation. Percentages are based on number of valid cases; For APOE 4 genotype, data was missing for 4 subjects.

*MMSE* Mini-Mental State Examination, *APOE* ε4 carriers of at least one apolipoprotein E ε4 allele, *T+* refers to p-tau ≥ 73.65 pg/ml, *A+* means Aβ42/40 ≤ 0.08, *HC* cognitively healthy controls, *ADRs* cognitively normal first-degree relatives of AD patients, *SCD* subjective cognitive decline, *MCI* mild cognitive impairment, *DAT* mild dementia of the Alzheimer’s type.

### Multivariate analysis of inflammatory markers reveals four components

The PCA on the full panel of 16 inflammatory markers revealed four components with eigenvalues > 1, explaining in total about 70% of the variance. The PCA components and their rotated loadings are shown in Supplementary Fig. 2 and unrotated loadings are reported in Supplementary Table 1. The main component (PC1) explained ca. 22% of the variance (after rotation) and showed high positive loadings on sTyro3, sAXL, sTREM2, YKL-40 and C1q. The second component (PC2) explained 19% of the variance and showed high positive loadings on CRP, C4, Factor B, Factor H and IL-18. The third component (PC3) explained 16% of variance and showed higher positive factor loadings on IP-10, MCP-1, MIF and IL-6. Finally, the fourth component (PC4) explained 12% of variance and showed strongest positive loadings on C3 and C3b. Results are summarized in Fig. 1B. We note that C1q, Factor H and MIF had loadings higher 0.4 on more than one factor. The covariance among all markers has been reported in [8] (Supplementary Material, Data-S1, AF-4).

### Inflammatory components relate to AD pathology and demographics

We first assessed how the inflammatory components related to Aβ42/40, *p*-tau and demographics (i.e., age, gender, BMI, education, vascular risk, APOE 4 genotype). Results are summarized in Fig. 1B and all correlations are shown in Supplementary Table 2. Higher PC1 was related to higher age (*r* = 0.281, *p* < 0.001), higher levels of p-tau181 (*r* = 0.627, *p* < 0.001), more pathological Aβ (lower Aβ42/40, *r* = −0.187, *p* = 0.001), APOE 4 genotype (*T* (289) = 2.96, *p* = 0.003) and lower BMI (*r* = −0.218, *p* < 0.001). When accounting for p-tau (see [8]), higher PC1 was related to less pathological Aβ (*r* = 0.326, *p* < 0.001). Higher PC2 was associated with higher BMI (*r* = 0.226, *p* < 0.001), higher vascular risk (rho = 0.145, *p* = 0.026) and male gender (T (293) = −4.39, *p* < 0.001). Higher PC3 was related to higher age (*r* = 0.126, *p* = 0.030) and higher vascular risk (rho = 0.144, *p* = 0.027). Finally, higher PC4 was related to higher BMI (*r* = 0.129, *p* = 0.027). None of the PCs was related to years of education (all *r* < 0.1, all *p* > 0.26).

### Inflammatory components relate to brain structural integrity at baseline

#### Associations with global (summary) brain measures

**Fig. 2:**
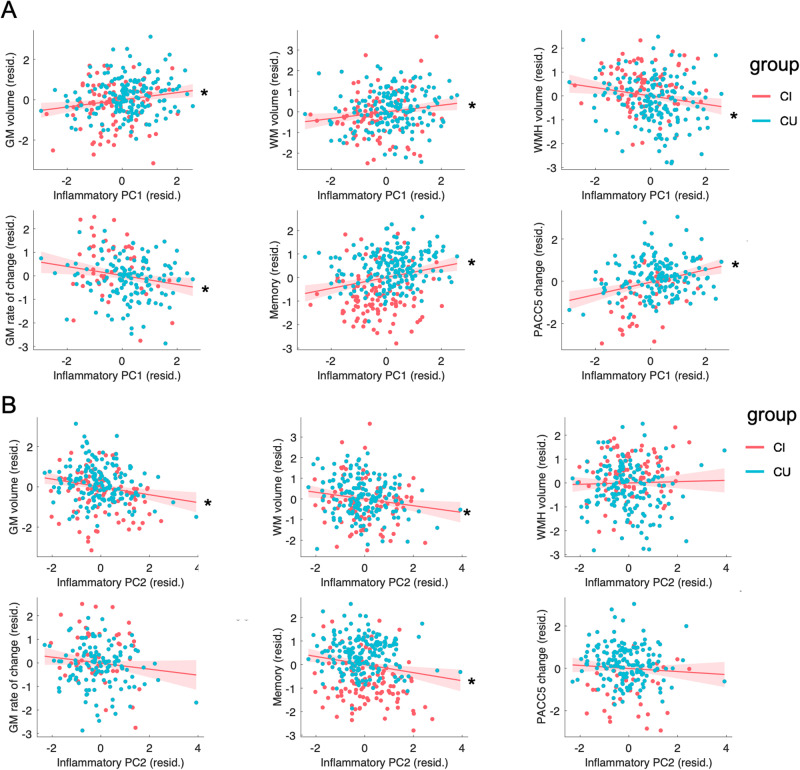
Associations of PC1 and PC2 with brain structural and cognitive measures. Scatter plots for the associations of PC1 (**A**) and PC2 (**B**) with global gray matter (GM) volume, global white matter (WM) volume, global white matter hyperintensity (WMH) volume, GM rate of change over follow-ups in AD vulnerable areas (first eigenvariate form PCA, high values denote faster shrinkage), memory performance at baseline and PACC5 change over follow-ups (slope extracted from LME models). Standardized residuals are shown after regressing out covariates (age, gender, p-tau181, Aß42/40 and ICV for brain measures). * denotes significant associations in the full regression models (Tables 2 and 3). CU cognitively unimpaired, CI cognitively impaired.

Results of the three regression models predicting global GM, WM and WMH volume by all four inflammatory components are presented in Table 2. For both global GM and WM volume, higher PC1 was related to higher volume and PC2 to lower volume (see also Fig. 2) with no significant effects of PC3 or PC4. When predicting WMH volume, higher PC1 was related to lower WMH volume with no significant effects of the other components. These results were similar when correcting for volume of the other image modalities. Furthermore, all results were consistent when additionally correcting BMI, vascular risk and APOE 4 genotype except for the effect of PC1 on WM volume, which was only marginal when adding APOE 4 genotype as covariate (*F*(1,221) = 2.9, *p* = 0.089).

**Table 2:** Regression models on brain structure predicted by inflammatory components.

Dependent	independent	*F*	Sig.	Partial eta2	*B*	SE	*T*
GM volume	age*	79.31	<0.001	0.242	−2.791	0.313	−8.906
	ptau181*	18.11	<0.001	0.068	−0.365	0.086	−4.256
	Aβ42/40	<1	0.737	<0.001	24.824	73.944	0.336
	ICV*	466.78	<0.001	0.653	0.322	0.015	21.605
	PC1*	**8.62**	**0.004**	**0.034**	**6.433**	**2.191**	**2.937**
	PC2*	**10.27**	**0.002**	**0.040**	**−5.420**	**1.692**	**−3.204**
	PC3	<1	0.923	<0.001	0.159	1.652	0.096
	PC4	<1	0.381	0.003	−1.434	1.634	−0.878
	gender*	10.11	0.002	0.039	14.752	4.640	3.179
WM volume	age*	66.47	<0.001	0.211	−3.318	0.407	−8.153
	ptau181	3.16	0.077	0.013	−0.198	0.111	−1.778
	Aβ42/40	<1	0.855	<0.001	17.540	95.999	0.183
	ICV*	283.76	<0.001	0.534	0.326	0.019	16.845
	PC1*	**6.77**	**0.010**	**0.027**	**7.401**	**2.844**	**2.602**
	PC2*	**7.01**	**0.009**	**0.027**	**−5.815**	**2.196**	**−2.648**
	PC3	0.29	0.588	0.001	−1.162	2.145	−0.542
	PC4	<1	0.991	<0.001	0.025	2.121	0.012
	gender	<1	0.926	<0.001	−0.559	6.024	−0.093
WMH volume	age*	29.26	<0.001	0.109	0.081	0.015	5.409
	ptau181	1.61	0.206	0.007	0.005	0.004	1.268
	Aβ42/40*	8.00	0.005	0.033	−10.004	3.517	−2.844
	ICV*	6.15	0.014	0.025	0.002	0.001	2.481
	PC1*	**6.51**	**0.011**	**0.027**	**−0.270**	**0.106**	**−2.551**
	PC2	<1	0.626	0.001	0.039	0.080	0.487
	PC3	2.54	0.113	0.011	0.125	0.079	1.593
	PC4	1.51	0.220	0.006	0.095	0.078	1.230
	gender	3.20	0.075	0.013	0.408	0.228	1.788
rate of GM change	age	0.84	0.360	0.005	0.002	0.002	0.919
	ptau181*	18.21	<0.001	0.100	0.002	0.001	4.268
	Aβ42/40*	4.16	0.043	0.025	−0.862	0.423	−2.039
	ICV	<1	0.618	0.002	4.5E−05	9E−05	0.500
	PC1*	**6.37**	**0.013**	**0.037**	**−0.032**	**0.012**	**−2.523**
	PC2	2.53	0.114	0.015	−0.016	0.010	−1.590
	PC3	<1	0.819	<0.001	−0.002	0.009	−0.229
	PC4	<1	0.393	0.004	0.008	0.010	0.857
	gender	<1	0.511	0.003	−0.018	0.028	−0.659

Regression models tested whether inflammatory components (PC1−4) derived from a PCA predicted total gray matter (GM) volume (*N* = 258), total white matter (WM) volume (*N* = 258), white matter hyperintensity (WMH) volume (*N* = 249) or rate of GM change in AD-vulnerable areas (*N* = 174). Please note that higher values of this first eigenvariate of GM volume change denote faster atrophy. Significant (*) effects of interest are highlighted in bold. Additional covariates of no interest in all models included age, gender, intracranial volume (ICV), p-tau and Aβ42/40. Additional regression plots are shown in Fig. 2.

Relationships of PC1/PC2 with brain structure were not moderated by clinical-pathological disease stage (see Supplementary Results) and remained similar when excluding the DAT patients. Supplementary partial correlations (Supplementary Table 3) of individual inflammatory markers loading on PC1 and PC2 showed strongest positive associations with brain structure for sTyro and sAXL (loading on PC1) and strongest negative associations for CRP, IL-18, Factor B and H (loading on PC2).

#### Spatial pattern of local (voxel-wise) association with brain volume

**Fig. 3:**
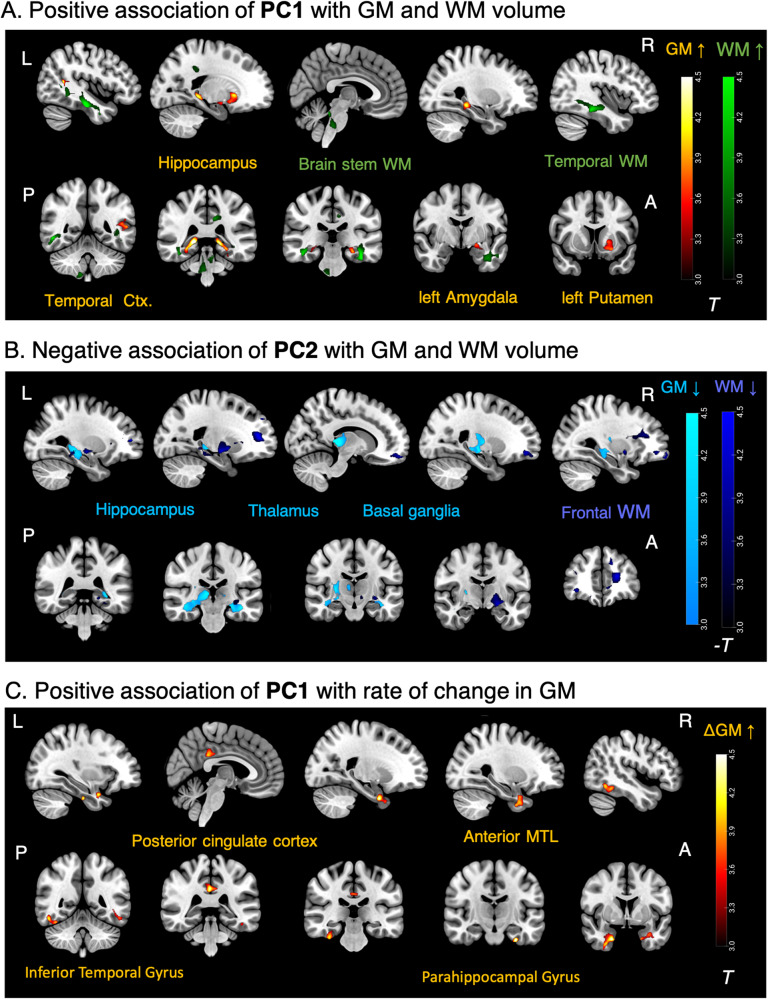
Whole-brain voxel-wise regression of brain volumes and rate of volume change on inflammatory components. **A** Higher levels of inflammatory component PC1 were related to higher GM volume (orange) at baseline in bilateral hippocampus, left amygdala and putamen and higher WM volume (green) in temporal regions and brain stem. *N* = 258. **B** Higher levels of the inflammatory component PC2 were related to lower GM volume (light blue) at baseline in bilateral hippocampus, right thalamus and right putamen and lower WM volume (dark blue) in frontal and other regions. *N* = 258. **C** Levels of the PC1 were positively related to rate of change in GM volume (i.e., less/slower atrophy over follow-ups) in anterior medial temporal lobe, posterior inferior temporal cortex and posterior cingulate cortex (orange). *N* = 174. Results are depicted at *p* < 0.05 (FWE, cluster-level, cluster forming voxel-level threshold *p* = 0.001). See Supplementary Fig. 3 for results at voxel-level threshold *p* = 0.005. All analyses were accounting for age, gender, ICV, p-tau181, Aβ42/40. L Left, R Right, A Anterior, P Posterior.

We further examined the spatial pattern of whole-brain voxel-wise associations of PC1 and PC2 with maps of GM, WM and WMH volume via VBM (Fig. 3A, B). PC1 was related to higher GM volume in hippocampus, amygdala and basal ganglia (putamen), as well as to higher WM volume in temporal areas and in brain stem (Fig. 3A). In contrast, PC2 was related to lower GM volume in hippocampus, thalamus and basal ganglia as well as lower WM volume mainly in frontal areas (Fig. 3B). Voxel-wise regressions of WMH lesion probability on both inflammatory components were not significant when accounting for p-tau181 and Aβ42/40.

### Inflammatory components relate to longitudinal GM volume change

#### Associations with summary measure of longitudinal atrophy

We first derived a summary measure of GM atrophy in AD-related areas by PCA and the first component of rate of GM volume change was highly loading on voxels in the anterior medial temporal lobe, posterior temporal cortex and posterior-midline regions (Fig. 1A). Higher PC1 predicted slower volume changes (i.e. less atrophy) over time (Table 2; Fig. 2A). There were no significant effects of higher order inflammatory components. We also observed a significant interaction between PC1 and clinical-pathological AD stage on GM change in AD-related regions (see Supplementary results), where higher PC1 values were related to slower volume change particularly in the CI A+ subgroup. Supplementary partial correlations of individual inflammatory markers loading on PC1 with rate of GM volume changes in AD-related areas showed strongest positive associations for sTyro and sAXL (Supplementary Table 3).

We additionally performed the same regression analysis as above using whole-brain averaged rate of GM volume change which revealed no significant associations to any inflammatory PC (all *p* > 0.27, all *F* < 1.3) neither to age or AD biomarkers (all *p* > 0.14., all *F* < 2.2) suggesting that effects of PC1 on atrophy rates are region specific.

#### Spatial pattern of local (voxel-wise) association with rate of GM volume change

VBM whole-brain analyses showed that higher PC1 values were positively related to GM volume change (i.e., showing less shrinkage) specifically in the anterior medial temporal lobe including entorhinal cortex and temporal pole, but also posterior inferior temporal and posterior cingulate cortex (Fig. 3C), confirming our findings above on the summary measure of GM change in AD-vulnerable regions.

### Inflammatory components relate to cognition

Results of regression analyses assessing the effects of all four inflammatory components on memory at baseline and on cognitive changes (PACC5) are summarized in Table 3. Higher PC1 was related to better memory performance at baseline, whereas higher PC2 was related to lower memory performance at baseline (see also Fig. 2). There were no significant effects of PC3 or PC4 on memory. Supplementary analyses on the other cognitive domain scores showed similar (but less strong) associations of PC1 and PC2 with other cognitive domains (Supplementary Table 4).

**Table 3:** Regression model for memory and LME for PACC5 change predicted by inflammatory components.

Dependent	independent	*F*	*P*	Partial eta2	*B*	SE	*T*
memory	age*	26.71	<0.001	0.085	−0.042	0.008	−5.168
	ptau181*	27.23	<0.001	0.087	−0.012	0.002	−5.219
	Aβ42/40*	33.88	<0.001	0.106	11.540	1.983	5.820
	PC1*	**14.86**	**<0.001**	**0.049**	**0.230**	**0.060**	**3.855**
	PC2*	**8.89**	**0.003**	**0.030**	**−0.133**	**0.045**	**−2.982**
	PC3	1.28	0.258	0.004	−0.050	0.044	−1.133
	PC4	<1	0.578	0.001	0.024	0.043	0.558
	gender	<1	0.467	0.002	0.065	0.089	0.729

Dependent	independent		*P*	CI	Estimate	SE	*T*
PACC5	time*		<0.001	0.36 – 1.23	0.8	0.221	3.610
	age*		<0.001	−0.07 – −0.03	−0.05	0.012	−4.160
	gender*		0.004	−0.60 – −0.11	−0.36	0.125	−2.860
	Aβ42/40*		0.017	0.03 – 0.31	0.17	0.072	2.381
	ptau181*		0.003	−0.45 – −0.09	−0.27	0.091	−2.929
	PC1*		0.019	0.03 – 0.36	0.2	0.084	2.351
	PC2*		0.044	−0.25 – −0.00	−0.13	0.063	−2.016
	PC3		0.074	−0.22 – 0.01	−0.11	0.060	−1.789
	PC4		0.544	−0.16 – 0.08	−0.04	0.060	−0.608
	time × age*		<0.001	−0.02 – −0.01	−0.01	0.003	−3.723
	time × gender		0.802	−0.07 – 0.06	−0.01	0.033	−0.251
	time × Aβ42/40		0.182	−0.01 – 0.06	0.02	0.019	1.335
	time × ptau181*		<0.001	−0.16 – −0.06	−0.11	0.025	−4.230
	**time** × PC1*		**<0.001**	**0.04 – 0.13**	**0.08**	**0.023**	**3.552**
	time × PC2		0.476	−0.04 – 0.02	−0.01	0.017	−0.713
	time × PC3		0.495	−0.04 – 0.02	−0.01	0.016	−0.683
	time × PC4		0.452	−0.05 – 0.02	−0.01	0.017	−0.754

A first regression model tested whether inflammatory components (PC1−4) predicted episodic memory at baseline (*N* = 295). For effects on longitudinal cognitive change, an LME (linear mixed effects model) was run to predict PACC5 scores in subjects with at least 3 time points (*N* = 204, *N* observations = 856). Significant (*) effects of interest are highlighted in bold. Additional covariates of no interest in all models included age, gender, p-tau181, Aβ42/40. Supplementary regression plots are shown in Fig. 2. *CI* confidence interval derived by LME.

With respect to cognitive decline, LME analyses showed that higher PC1 at baseline was also related to less decline over follow-ups in PACC5 (time × PC1 interaction) with no significant effects of the other PCs.

These results on cognition were consistent when correcting for BMI, vascular risk, and APOE4 genotype. Relationships of PC1/PC2 with cognition were not moderated by disease stage (see Supplementary Results) and remained significant when excluding the DAT patients. Supplementary partial correlations of individual inflammatory markers loading on PC1 and PC2 with cognitive measures showed strongest positive associations for sTyro, sAXL and sTREM2, and strongest negative associations for CRP and Factor B/H (Supplementary Table 3).

### Effect replication in the F.ACE cohort

A PCA in the F.ACE cohort revealed similar components PC1 and PC2 as in DELCODE (Supplementary Fig. 2) that showed similar associations to demographics and AD pathology. In the F.ACE cohort, PC1 was also related to better memory at baseline and less cognitive decline over time (if controlling for p-tau only) as well as higher thickness in AD vulnerable regions. PC2 was not related to cognition but showed negative associations with thickness in AD vulnerable areas. The detailed analyses are described in the Supplementary Material (Supplementary Table 5-8).

## Discussion

Our study revealed different inflammatory signatures based on immune-related CSF biomarkers. Specifically, a PCA on the 16 inflammatory markers in DELCODE revealed a first component (PC1) that related to preserved brain structure and cognition, and a second component (PC2) that related to reduced brain structure and cognition. The two other components (PC3 and PC4) were not linked to brain structure or cognition. Overall, this suggests that certain markers that likely reflect different underlying inflammatory or immune processes might be beneficial or detrimental with regard to brain health and cognitive function.

PC1 was mostly weighted by soluble receptors sTREM2, sAXL and sTyro, as well as YKL-40 and C1q. PC1 increased with older age and more tau pathology, but also related to lower pathological Aβ levels for a given degree of tau pathology. When adjusting for AD pathology, this inflammatory component was related to higher GM and WM volume (particularly in AD vulnerable areas), less WM lesions, better memory at baseline and less cognitive decline. The main contributors to these positive associations were sTyro3 and sAXL, which corroborates our previous findings in the same cohort [8], and weaker contributions were seen for sTREM2. Our novel longitudinal imaging analysis further showed that higher PC1 levels at baseline predicted less atrophy over time in an AD vulnerable temporal lobe atrophy network. Although the results could also be explained by swelling, the association of PC1 with reduced cognitive decline over time favors a protective role of this inflammation-related component, and also makes it unlikely that results are simply explained by higher brain reserve. Furthermore, our novel data revealed beneficial effects of PC1 on WM integrity represented by higher WM volume and fewer WM lesions even when adjusting for GM volume, which would further support that PC1 relates to brain protection. In the F.ACE cohort, we could replicate the PC1 signature that related to better cognition as well as higher GM integrity in AD signature regions.

TAM (Tyro3, AXL, Mertk) receptors are cell surface receptors on glia cells and neurons transmitting signals from the extracellular space to the cytoplasm and nucleus (for regional expression profiles, see [8]). TAM receptor signaling modulates neurogenesis, neuronal migration, synaptic plasticity, and vascular remodeling (e.g., [35]), and it controls microglial activation, phagocytosis, myelination, and peripheral nerve repair (for review, see [36, 37]). Moreover, TAM receptors are critically involved in phagocytosis of apoptotic cells [38] and anti-inflammatory signaling, thereby contributing to the maintenance of brain homeostasis and to clearance of pathological protein aggregates (e.g., [36, 39–41]. It was demonstrated [38] that the microglial response to brain damage is TAM-regulated, where microglia employ TAM receptors to seek out and engulf Aβ plaques [39] (which relies in part on the receptor TREM2) and their genetic ablation also increased cerebral amyloid angiopathy. While our associations between TAM receptor levels and Aβ pathology were cross-sectional, one could hypothesize that TAM receptor signaling is activated as a “damage response” and relates to protection of neurons and vessels due to improved clearance of pathological proteins such as Aβ [41], which is represented by preserved volume and reduced vascular lesions. This would be further supported by recent longitudinal PET data, showing that increased TAM receptor levels and their ligand (Gas6) in CSF predicted slower Aβ and tau accumulation in A+ and T+ non-demented older adults, respectively [42].

TREM2 has also been implicated in phagocytosis of apoptotic cells, cellular debris, lipoproteins, Aβ, and bacteria as well as in anti-inflammatory signaling and promotion of cell survival (for review see [43, 44]). Microglial TREM2 is crucial for the stimulation of disease associated microglia (DAMs), characterized by an upregulation of Axl, which are seen in association with neurodegeneration and which are thought to play a protective role in AD due to their role in phagocytosis [43, 45, 46]. Previous longitudinal cognitive and neuroimaging data in humans also support that TREM2 signaling might be related to neuroprotective effects [7, 47, 48].

In addition, C1q and YKL-40 highly loaded on the first inflammatory component, with C1q also loading on PC2. Their correlations with TREM2, sAXL and sTyro3 were moderately strong, suggesting that these markers increase together in relation to aging and neurodegeneration. However, YKL-40 and C1q were not individually related to brain structure or cognition, which does not support neuroprotective effects of these markers in particular. C1q is the recognition component that initiates the classical complement cascade, as part of the innate immune system, and to our knowledge no other studies have related C1q in CSF with brain structure in aging or AD yet. Recently, one meta-analysis showed an increased complement pathway activity in AD, which was mostly observed by elevated CSF clusterin concentrations [49]. YKL-40 has been involved in the astrocytic response to modulate neuroinflammation [50] and CSF levels of YKL-40 have been associated with GM volume as well as p-tau levels in a non-linear pattern [9], suggesting that increasing YKL-40 levels might relate to higher or reduced brain volume depending on the AD-related disease stage [51]. In our study, the observed associations of inflammatory components with brain structure or cognition at baseline did not differ by the clinical-pathological stage defined by AD biomarker levels and cognitive impairment. However, when predicting atrophy over time, positive associations of PC1 were particularly observed in the group of A + MCI and dementia patients, which suggests that beneficial effects are strongest when AD pathological changes and clinical impairment are already present. However, we note that this analysis was limited by the small size of subgroups and needs further replication in larger samples. Considering that TREM2, Tyro3 and AXL are upregulated in activated microglia including DAMs [43, 45, 52, 53], YKL-40 is a marker for activated astrocytes [50] and C1q may induce astrocytes activation [54], this suggests that PC1 might be reflect a beneficial inflammatory response related to DAM2 or astroglia activation as a response to brain damage to preserve brain structure by anti-inflammatory clearance mechanisms [41].

The second inflammatory component showed the opposite pattern compared to PC1, i.e. negative associations with GM (especially in hippocampus, basal ganglia and thalamus) and WM volume (especially in frontal areas) as well as with memory at baseline. PC2 showed highest positive loadings by the proinflammatory signaling molecules CRP and IL-18 as well as complement factors H, B and C4. CRP is a downstream product of the acute phase response and an activator of the complement system [55], and Factor H and Factor B are involved in the regulation of the complement system [56, 57]. When activated, the complement system causes the release of pro-inflammatory cytokines [58], such as IL-18. Higher PC2 levels were further related to higher vascular risk and BMI. Previous studies that assessed peripheral levels of CRP in blood and another proinflammatory marker, IL-6, have also reported negative associations with GM volume and WM volume in older adults [13–15] and even middle-aged adults [16]. Moreover, higher levels of these markers were related to higher BMI [16] suggesting that obesity may be a source of the inflammation. Blood levels of CRP and IL-6 have been also associated with future development of AD and vascular dementia (e.g., [59]). Although, in our study, PC2 was not predictive of cognitive change over several years, CRP itself was related to cognitive decline confirming the aforementioned earlier studies that measured CRP in blood. PC2 was, however, not related to WM lesions. In this regard, previous studies on peripheral CRP remain also inconclusive (e.g., [13, 14, 17, 28]). Finally, PC2 was also increased in males (even when correcting for BMI and vascular risk). Other past studies on peripheral CRP levels reported gender differences but in the opposite direction, with higher blood CRP levels in females (e.g., [60]). While it remains open what explains our observed differences in PC2 related to gender, we covaried for gender in all of our analyses. In the F.ACE cohort (which lacked IL-6 measures), a highly similar PC2 was derived that was also related to male sex, higher BMI as well as lower GM thickness in AD signature regions. However, PC2 was not related to worse memory. In summary, PC2 may be related to the release of pro-inflammatory cytokines that contribute to brain damage, but the mechanism underlying the concordant increases of these markers remains unclear.

One strength of our study is the unique and comprehensive set of different inflammation-related markers that was measured in a large sample enriched in older adults at risk of development of AD (such as SCD) in combination with longitudinal cognitive and neuroimaging data. Based on the view of a cellular phase of AD [4], understanding the disease requires unveiling the complex cellular interactions including inflammation. Accordingly, our study is one of the few that assessed inflammatory signatures using multivariate methods, rather than analyzing inflammatory markers separately and our combination of markers into signatures revealed consistent associations with brain and cognitive outcomes.

However, our study has some limitations. While we included longitudinal cognitive and atrophy data, inflammatory markers and AD biomarkers were only measured at baseline. Thus, the temporal process and ordering of events remains unclear (e.g., whether higher TAM receptor levels predict reduced Aβ accumulation over time [42]). Furthermore, there might be inter-individual variability in certain physiological phenomena, such as rates of CSF production, and rates of CSF clearance that could lead to differences in mean CSF protein levels and so far there are no established reference marker for inflammatory biomarkers. However, we note that our results largely remained consistent when controlling for CSF volume or global marker levels [34]. Finally, although we determined groups of inflammatory markers that were related to brain structure and cognition, the underlying processes that link these markers remain to be studied further and there are also several other relevant proteins that were not measured here.

## Conclusion

In summary, our study identified distinct inflammatory biomarker signatures, reflected by markers that likely increase together in the context of AD or vascular risk, which seem to represent either beneficial or detrimental inflammatory processes with regard to brain integrity. The first biomarker signature was orchestrated by soluble TAM receptors and sTREM2 and may represent protective responses in the brain by stimulation of DAMs, supporting their role in phagocytic clearance and tissue repair. Our findings further highlight that inflammation represents a potential intervention target, as well as readout in clinical trials aside from the classical AD hallmarks [41]. Furthermore, inflammatory markers might be important for stratification of participants in clinical AD trials, especially if atrophy measures or cognitive decline are used as outcome measures. Future longitudinal studies are still needed to elucidate their role in the course of disease progression, to subsequently use them as an intervention target or for stratification. Moreover, future studies that use microglia- or astroglia-specific PET tracers would further allow to study the regional pattern of microglia and astroglia activation [61].

### Supplementary information

Supplementary Material

## Data Availability

There are restrictions to the availability of data from the DELCODE study as well as from F.ACE due to study regulations. For details and contact, please see https://www.dzne.de/en/research/studies/clinical-studies/delcode/ or contact F.ACE (https://www.fundacioace.com/en/contact-us.html), respectively.
