# Supplementary material for: Different inflammatory signatures based on CSF biomarkers relate to preserved or diminished brain structure and cognition
Source: Mol Psychiatry. 2024 Jan 12;29(4):992–1004. doi: 10.1038/s41380-023-02387-3 (PMC11176056; doi:10.1038/s41380-023-02387-3)
Supplement: Supplementary file 1 — Supplementary Material [file 41380_2023_2387_MOESM1_ESM.docx]

Supplementary

# Supplementary Methods

## MRI image acquisition

MRI data were acquired with Siemens scanners (3 TIM Trio systems, 4 Verio systems, one Skyra and one Prisma system) at 10 different scanning sites. The current analysis was performed using the T1-weighted images (MPRAGE, 3D GRAPPA PAT 2, 1 mm3 isotropic, in-plane resolution = 256x256px, 192 slices, sagittal, ~5min, TR = 2500ms, TE = 4.33ms, TI = 110ms, FA = 7°) and the FLAIR images (TR = 5000ms, TE = 39 ms, inversion time = 1.8s, voxel size = 1mm3 isotropic, in-plane resolution = 256x256px, 192 slices). Standard operating procedures and quality assessment were provided by the DZNE imaging network. Quality assessment yielded to the exclusion of one T1-weighted MPRAGE (status “unusable”).

# Supplementary Data and Results

## Analysis of clinical-pathological stages in DELCODE

We defined the clinical-pathological stages of AD spectrum by cognitive and biomarker staging of AD pathology according to [1], where individuals with abnormal Aβ levels are in the Alzheimer’s continuum. We defined three groups with increasing risk of AD: i) cognitively unimpaired with normal AD biomarkers: CU A-T- (N = 133), ii) cognitively unimpaired with abnormal Aβ levels: CU A+ (N = 51), iii) cognitively impaired with abnormal Aβ levels: CI A+ (N = 74). Thirty-seven subjects did not fit any group criterion. There were no significant interactions between PC1 or PC2 with clinical-pathological AD stages (CU A-T-, CU A+, CI A+) on any of the brain measures at baseline (all p>0.3, all F<1.2).

With regard to longitudinal GM change, we observed a significant interaction (i.e., moderation) between PC1 and clinical-pathological AD stage on GM rate of change in AD-related regions (F (2,149) = 5.48, p = 0.005, adjusting for p-tau181). Using post-hoc tests, higher values of PC1 were related to slower GM change only in the CI A+ subgroup (F (1,27) = 7.36, p = 0.011) but not CU A-T- subgroup (F (1,90) <1, p>0.4) or CU A+ subgroup (F (1,24) <1, p>0.9) while controlling for p-tau levels. However, we note that groups were small for the subsample with longitudinal atrophy data (N: CU A-T- = 96, CU A+ = 30, CI A+ = 33).

When testing for moderation of associations between PC1 and cognition by disease stage, we did not find any significant interactions between PC1 and clinical-pathological AD disease stage on memory at baseline (F(2,249) =1.02, p = 0.36) or on change in PACC5 (F(2,171) <1, p>0.4).

## Sensitivity Analyses in DELCODE

CSF biomarker concentrations may be influenced by non-disease related mechanisms which vary between individuals, such as CSF production and clearance rates. Adjustment by reference proteins has been shown to improve the diagnostic accuracy of CSF Alzheimer’s disease biomarkers such as p-tau181 and Aβ42. Aβ40 has been established as a reference protein for Aβ42 as normalization increased expected associations with Aβ PET (e.g. [2, 3]). For p-tau181, normalization by Aβ40, NTRK3, NTRK2 or mean CSF marker levels improved the accuracy of predicting PET-based tau burden [2, 3]. For inflammatory biomarkers, no reference proteins have been established yet.

In order to test how our findings would be influenced by different adjustments that aim to account for interindividual differences in global marker levels, we ran models to account for i) CSF volume ii) global inflammatory marker levels and ii) Aβ40.

In addition, we also tested how results would change when we include diagnostic group into our models (iv).

### i) CSF volume

All results for PC1 remained significant when accounting for CSV volume. For PC2 associations with memory were only marginal (F(1,248)=2.85, p=.093, T=-1.69, Beta=-0.056, error=0.048) when CSF volume was included as a regressor to the models. All other effects of PC2 remained significant (i.e., negative associations with global GM and WM volume).

### ii) Global inflammatory marker levels

Global inflammatory marker levels were derived as the average across all 16 markers after Z-standardization. All 4 inflammatory PCs as well as Aβ40 and p-tau181 were positively (moderately) correlated with our global inflammatory marker score (PC1: r=.56, PC2: r=.58, PC3: r=.49 PC4: r=.33, p-tau: r=.38, Aβ40: r=.44; all p-values < 0.001) and these inter-marker correlations were similar or even slightly stronger within the A-T- cognitively unimpaired group (N=133). Moreover the global marker score did not differ between clinical groups (F(4,290)=1.9, p=0.11). When adjusting for global inflammatory marker levels, results for PC1 and PC2 remain significant, except for the negative association of PC1 with longitudinal atrophy that became marginal (F(1,167)=3.43, p = 0.06, T=-1.85, Beta=-0.026, error=0.014). Due to high collinearity of global marker levels with all 4 PCs, these models were run separately for each PC. Global marker levels were not significantly predicting any outcome measure in these regression models except for a negative association with memory, as opposed to the effect of PC1 that predicted better memory in the same model.

### iii) Aβ40

When adding Aβ40 as a regressor to our models (that included already p-tau181 and Aβ42/40) all results remain significant for PC2. PC1 remained a significant predictor of higher global gray matter volume (F(1,247)=7.1, p=0.008; T=2.7, Beta=6.94, error=2.60) and predicted less cognitive decline over follow-ups (F(1,190)=9.2, p=0.003, T=3.0, Beta=0.0021, error=0.0007). However, the association of PC1 with global WM volume was only marginal (F(1,248)=2.85, p=0.09, T=1.7, Beta=5.67, error=3.37) and the association with white matter hyperintensities (F(1,239)<1, p>0.2) and volume change was not significant anymore (F(1,163)<1, p>0.2). Results were similar in the same model when excluding Aβ42/40 as covariate. We note, that Aβ40 was strongly correlated with PC1 (r=.72), which leads to collinearity of both regressors in the regression models. Aβ40 also differed between diagnostic groups (F(4,290)=2.4, p=.049) due to significant reductions in SCD and MCI and marginal reductions in DAT patients relative to the control group suggesting that it does not only reflect non-disease related physiological variability in global marker levels.

### iv) Clinical Group

We also tested how the results would change when adding diagnostic group (HC/ADR, SCD, MCI, DAT) as a factor to the regression models. As expected, in all models clinical group was a significant predictor of the cognitive or brain structural outcome measures (all F>3, all p<0.03). When including clinical group into the model, the effect of PC1 on global GM volume was marginal (F(1,245)=3.02, p=0.08, T=1.74, Beta=3.90, error=2.24) and the negative association of PC2 remained significant (F(1,245)=9.36 , p=0.002, T=-3.06, Beta=-5.05, error=1.65). With respect to global WM volume the effect of PC1 was not significant (F(1,245)=2.59, p=0.11, T=1.61, Beta=4.76, error=2.96) but higher PC2 remained related to reduced WM volume (F(1,245)=6.62, p=0.01, T=-2.57, Beta=-5.59, error=2.17). Neither PC1 (F(1,236)=1.88, p=0.17) nor PC2 (F(1,236)<1, p>0.80) showed a significant effect on WMH volume when clinical group was included into the model.

With respect to memory performance at baseline, the effect of PC1 on memory was not significant (F(1, 283)<1, p>0.8, T<1) when including diagnostic group to the model but PC2 was still related to worse memory (F(1,283)=6.60, p=0.01, T=-2.57, Beta=-0.079, error=0.031). However, PC1 remained positively related to cognitive change in PACC5 (F(1,188)=5.52, p=0.02, T=2.35, Beta=0.0014, error=0.0006).

## Replication analyses in F.ACE

### F.ACE cohort and general overview of analyses

To test if our main findings discovered in DELCODE could be replicated in an independent cohort, we used the EU-JPND funded PREADAPT project comprising a cohort of SCD and MCI subjects (N = 184) of the Fundacı´o ACE (F.ACE) Alzheimer Center, Barcelona [4, 5]. For replication analysis we focused on the 2 major inflammatory components identified in DELCODE: PC1 and PC2. The markers that were mainly loaded on these components in DELCODE were sTyro3, sAXL, sTREM2, YKL-40, C1q, MIF, CRP, C4, Factor B, Factor H and IL-18. Except IL-18, all markers were also available in F.ACE.

In summary, we first performed a PCA, which also revealed two principal components similar to PC1 and PC2 in DELCODE. Based on our findings in DELCODE, we then assessed how these inflammatory components in F.ACE related to 1) baseline global and regional measures of GM structure (focusing on AD-vulnerable regions identified in DELCODE) derived from FreeSurfer segmentation of the T1-images 2) memory or global cognition at baseline 3) cognitive decline over time. As in DELCODE, we ran individual multiple regression models for each brain or cognitive measure including both inflammatory PCs as predictors and included age, gender, Aβ42, p-tau and assay type (as well as the interaction of assay with AD biomarkers) as covariates into the models. For models on FreeSurfer volumetric measures (i.e. for hippocampus, basal ganglia and whole brain volume), we also included total intracranial volume (ICV) as a covariate (see below).

Use of data and biomaterial of the F.ACE cohort for the work described in this manuscript was approved by the Ethical Committee of the Hospital Clinic I Provincial de Barcelona (HCB/2014/0494, HCB/2016/0571, HCB/2016/0835, HCB/2017/0125 and HCB/2018/0333). Protocols of F.ACE had been designed in agreement with the indications of the Sociedad Espanola de Neurologıa ([www.sen](http://www.sen).es), according to the current regulations for the use of clinical data and biological material and surplus of the assisted process for the biomedical research of neurodegenerative diseases. General information on the F.ACE cohort criteria and procedures has been described elsewhere [4, 6, 7].

The sample analyzed here included 56 SCD and 128 MCI subjects who all had available MRI data within 6 months of the baseline CSF measures. Supplementary Table 5 summarizes sample characteristics of the F.ACE sample used here.

### F.ACE biomarker, MRI and cognitive data

Routine AD CSF markers in F.ACE were obtained by use of different immunoassays: Assay 1 (N = 97), manual ELISA (Innotest) and Assay 2 (N = 87), automated CLEIA assay (LUMIPULSE). Aβ42/40 ratio was only available for Assay 2 and thus for the main analyses we used Aβ42. The following study-specific cut-off values were used for F.ACE data: Assay 1: Aβ42: 676 pg/mL, p-tau-181: 58 pg/mL; Assay 2: Aβ42: 796 pg/mL, p-tau-181: 54 pg/mL. For A/T biomarker classification, we did not differentiate between assays, but categorized data according to the assay-specific cut-offs. Assay type was used as covariate in our analyses (see below). Out of 184 individuals, 43 were A+ and 47 T+.

In DELCODE, we found strongest associations of PC1 and PC2 with our memory composite (factor) score. In the F.ACE cohort, we similarly computed a memory score using a z-composite score of the Wechsler memory scale III immediate and delayed word list recall. In addition, our longitudinal cognitive analyses in DELCODE used the PACC5 score. Because of differences in study design, not all items required for PACC5 score calculation were available in F.ACE. We therefore constructed a similar composite using a z-composite score of Wechsler memory scale III immediate and delayed word list recall, semantic fluency (animals named in one minute) and the automatic inhibition subtest from Syndrom-Kurz-Test (time to complete), as done in [5]. Finally, we also assessed associations with a standard measure of global cognition that was available in both cohorts: the MMSE.

Regarding structural MRI data, T1-images at baseline were processed with Freesurfer 7.3.2 in F.ACE (<https://surfer>.nmr.mgh.harvard.edu/) using the recon-all pipeline. Our voxel-wise whole brain analyses in DELCODE on regional patterns of associations between PC1 and PC2 with and GM structure revealed specific associations in AD vulnerable areas covering the anterior, medial and lateral temporal lobe, the posterior cingulate cortex and basal ganglia/thalamus. In F.ACE we focused on these areas by creating i) a composite thickness measure of cortical regions covering the temporal lobe and posterior cingulate, ii) bilateral hippocampal volume and iii) mean volume score for basal ganglia and thalamus. For all subcortical volumetric measures, ICV was added in the analyses as covariate.

### Results

### (i) PCA

A PCA on the panel of 10 inflammatory markers in F.ACE revealed 2 components with eigenvalues > 1, explaining in total about 73% of the variance. The PCA components and their loadings are shown in Supplementary Figure 2B. The main component (PC1) explained ca. 41% of the variance (after rotation) and showed high positive loadings on sTyro3, sAXL, sTREM2, YKL-40, C1q and MIF. The second component (PC2) explained 19% of the variance and showed high positive loadings on CRP, C4, Factor B, Factor H. These components are largely identical to the PC1 and PC2 in DELCODE. Please note that in DELCODE, MIF also loaded on PC1 but had its highest weight on PC3.

### (ii) Relationship of inflammatory components to AD pathology and demographics

We first assessed how the inflammatory components related to Aβ42, p-tau and demographics (i.e., age, gender, BMI, APOE4 genotype). Higher PC1 was related to higher age (r = 0.353, p<0.001), higher levels of p-tau181 (r = 0.637, p<0.001), higher Aβ42 (r = .375, p = 0.001; but lower Aβ42/40: r = -0.403, p<0.001 in the subgroup of 87 participants with Assay2), and lower BMI (r = -.304, p<0.001). When accounting for p-tau, higher PC1 was still related to higher Aβ42 (r = 0.563, p<0.001) reflecting less pathological levels of Aβ. Higher PC2 was associated with higher BMI (r = 0.388, p<0.001), and male gender (T (182) = 3.82, p<0.001). This largely replicates our findings in DELCODE.

### (iii) Relationship of inflammatory components to brain structural integrity at baseline

In F.ACE, PC1 predicted higher GM thickness and PC2 lower GM thickness in the cortical AD signatures regions, when controlling for assay type and assay interaction with AD biomarkers (see Supplementary Table 6). There was no significant association with basal ganglia/thalamus or bilateral hippocampus volume (all p-values >0.10; all F-values < 2.6). However, we note that PC1 predicted bilateral hippocampus volume in a simpler model without assay type as covariate (F (1,175) = 4.1, p = 0.043). Moreover, when focusing on the small subsample measured with assay 2 and available Aβ42/40, PC1 also related to higher whole brain GM volume (F (1,79) = 5.4, p = 0.022) and marginally to higher hippocampal volume (F (1,79) = 3.3, p = 0.07), which replicates our findings in DELCODE.

### (iv) Relationship of inflammatory components to cognition

As in DELCODE we found that in F.ACE, PC1 predicted higher memory performance at baseline as well as better global cognition (MMSE) (see Supplementary Table 7) when controlling for p-tau and Aβ42 levels. However, PC2 was not significantly related to worse cognition in the F.ACE cohort. Regarding longitudinal cognitive decline, PC1 predicted less cognitive decline over time when controlling for p-tau levels (and for the p-tau x time interaction; see Supplementary Table 8, Model 1). Notably, also p-tau was negatively related to cognitive change in this model. However, when also adding Aβ42 into the model, none of the AD markers or inflammatory PCs related to cognitive change (see Supplementary Table 8, Model 2). However, we note that p-tau, PC1 and Aβ42 levels were correlated and no Aβ42/40 ratio was available for all participants in F.ACE, in contrast to DELCODE.

# Supplementary Figures


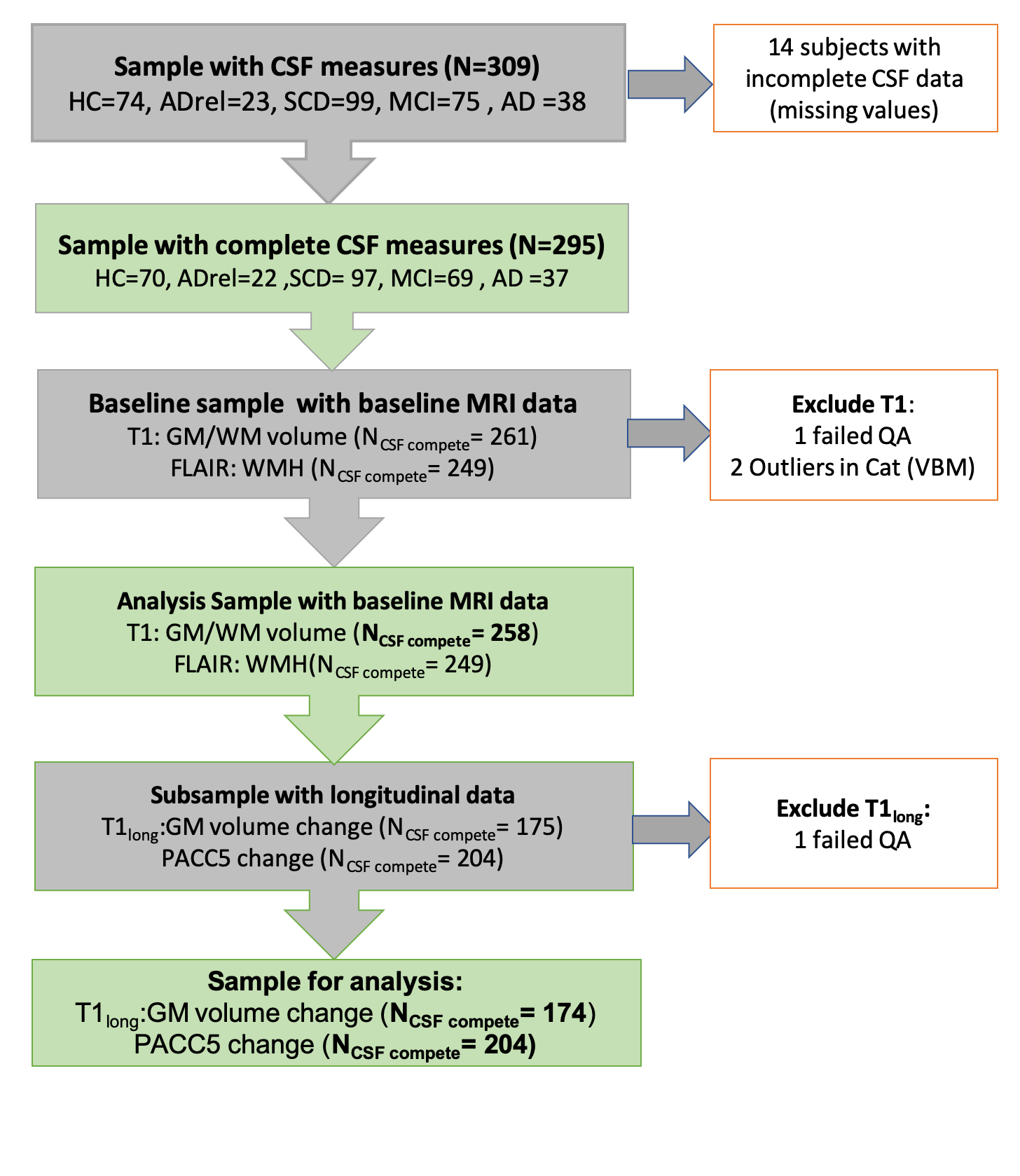


**Supplementary Figure 1. Flow chart Sample**. The flowchart shows the original sample of DELCODE subjects who received CSF sampling and the analyzed subsamples below. Only individuals with a complete panel of all 16 CSF inflammatory markers and ptau181 and Aß42/40 ratio were included in the study (N = 295). All of these had cognitive data at baseline. MRI T1 baseline data was available in 261 subjects and FLAIR images in 249 subjects. We excluded subjects with QA status “unusable” (T1: N = 1) and extreme outliers identified by the homogeneity check on segmented gray or white matter images in the CAT toolbox (N = 2). Longitudinal T1 data (3 follow-ups) were available on 175 subjects and cognitive follow-up (PACC5 score) in 204 subjects. One T1 follow up scan had QA status “unusable”.


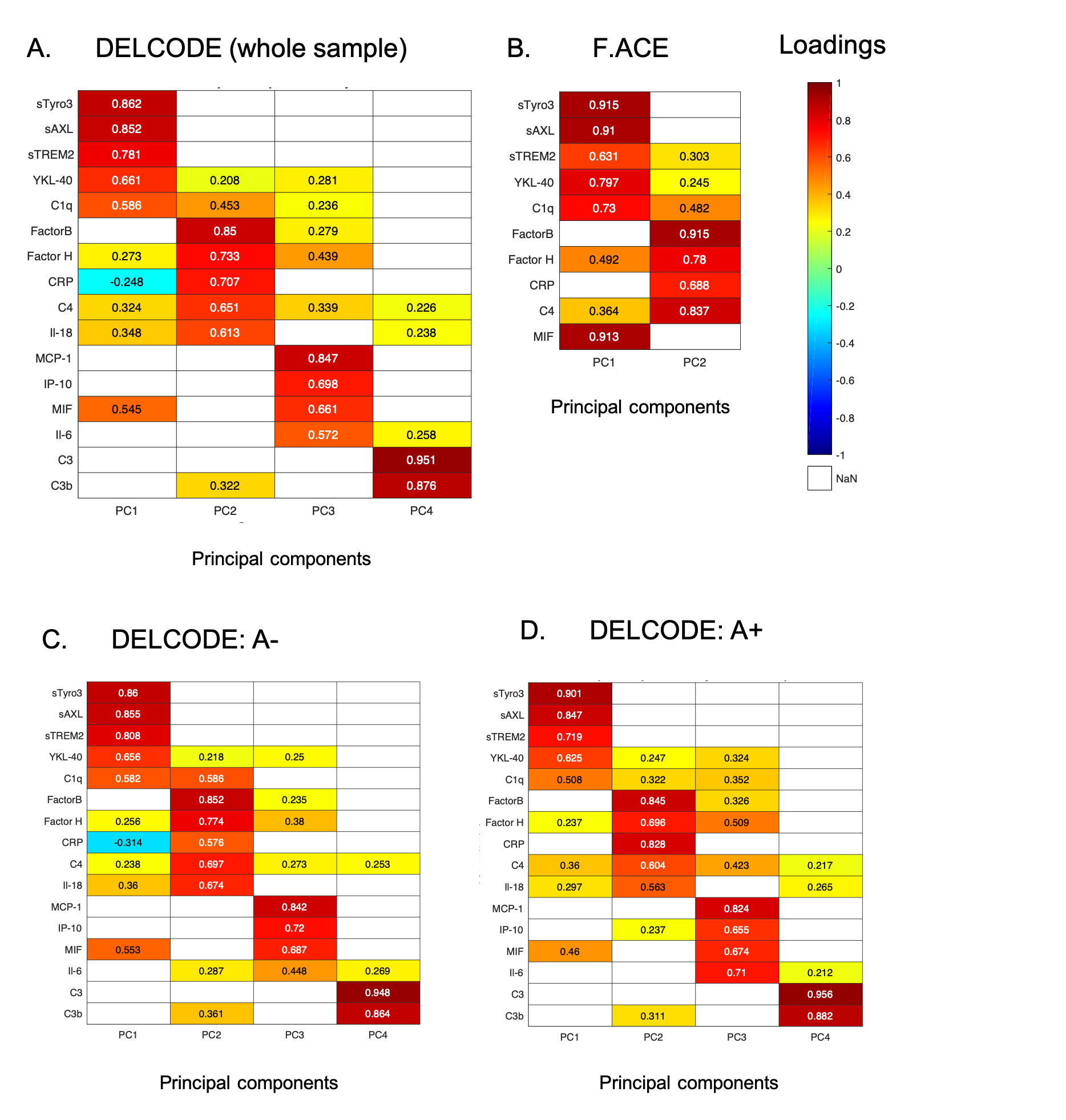


**Supplementary Figure 2** Principal component analysis on inflammatory markers in DELCODE and F.ACE. Heat map of the rotated loading matrix after PCA (and subsequent varimax rotation) on the inflammatory CSF markers showing the loadings (contributions) of each of the markers to the obtained component scores. **(A)** Principal components (PCs) and their loadings on individual markers in the DELCODE sample (N = 295). The CSF biomarker panel included 16 inflammatory markers. **(B)** PCs and their loadings derived from the F.ACE sample (N = 184). Please note that only 10 markers were available here (those that also loaded on PC1 and PC2 in DELCODE). IL-18 was not assessed in F.ACE. PC results for A- individuals **(C)** and A+ individuals **(D)** in DELCODE revealed relatively similar results. Loadings less than 0.2 were neglected for display purposes. Abbreviations: sTyro3: soluble tyrosine-protein kinase receptor; sAXL: soluble AXL Receptor Tyrosine Kinase (UFO); sTREM2: soluble Triggering Receptor Expressed On Myeloid Cells 2; YKL-40: Chitinase-3-like protein 1 (CHI3L1); C1q: complement component 1q; Factor B: complement factor B; Factor H: complement factor H; CRP: C-reactive protein; C4: complement component 4; Il-18: interleukin-18; MCP-1: monocyte chemotactic protein 1; IP-10: interferon gamma-induced protein 10; MIF: macrophage migration inhibitory factor; Il-6: interleukin-6; C3: complement component 3; C3b: complement component 3b.


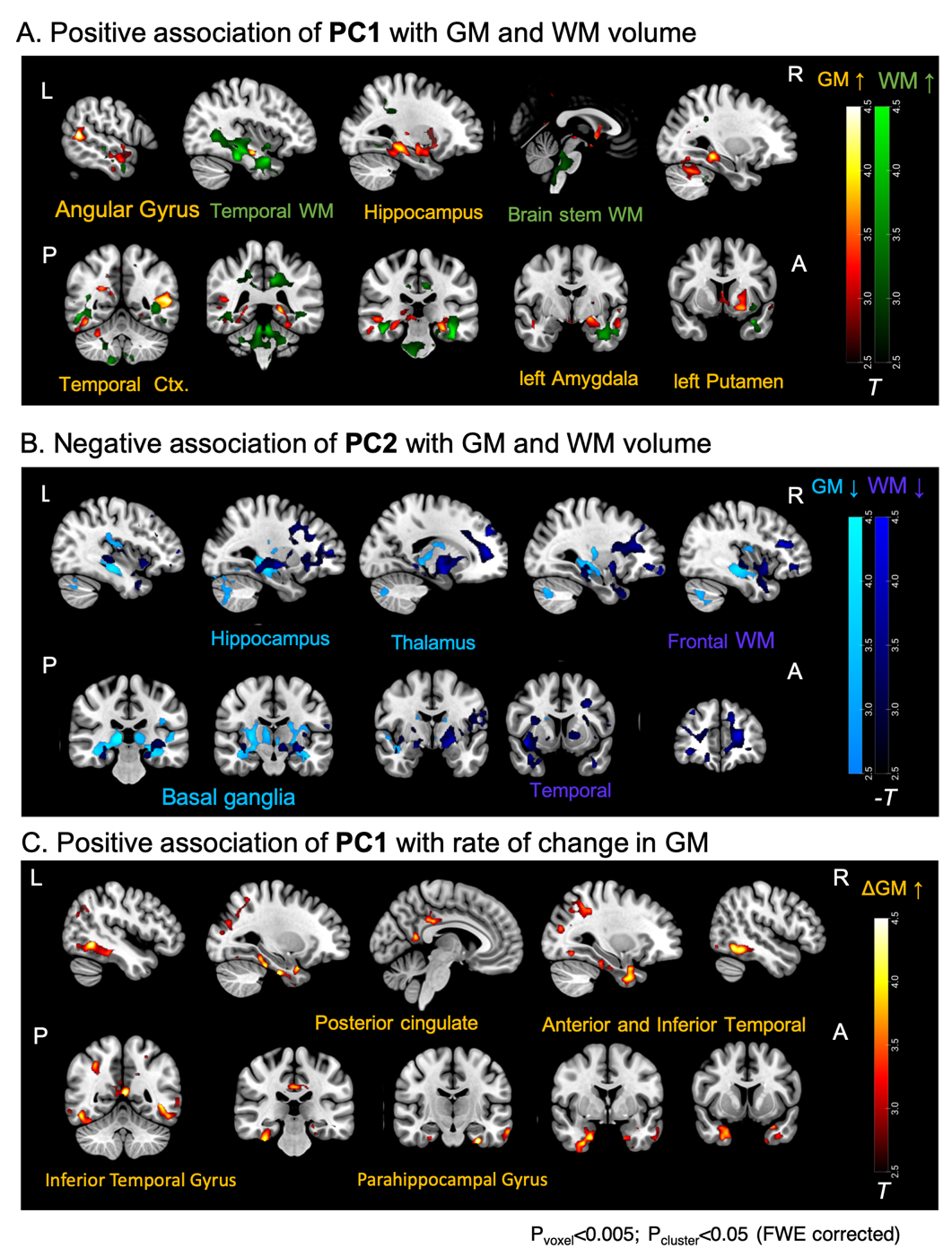


**Supplementary Figure 3.** Whole-brain voxel-wise regression of brain volumes and rate of volume change on inflammatory components at a more liberal cluster forming voxel-level threshold in the DELCODE sample. **(A)** Higher levels of inflammatory component PC1 were related to higher GM volume (orange) at baseline in hippocampus, amygdala, putamen, temporal cortex and angular gyrus. Higher PC1 was related to higher WM volume (green) in widespread temporal regions, frontal regions and brain stem. N = 258. **(B)** Higher levels of PC2 were related to lower GM volume (light blue) at baseline in hippocampus, thalamus, putamen and cerebellum and lower WM volume (dark blue) in frontal, temporal and other regions. N = 258. **(C)** Levels of the PC1 were positively related to rate of change in GM volume (i.e., less/slower atrophy) in anterior and posterior medial and lateral temporal lobe and posterior cingulate cortex and parietal regions (orange). N = 174. Results are depicted at p<0.05 (FWE, cluster-level, cluster forming voxel-level threshold p = 0.005). All analyses were accounting for age, gender, ICV, p-tau181, Aβ42/40. L=Left, R=Right, A=Anterior, P=Posterior


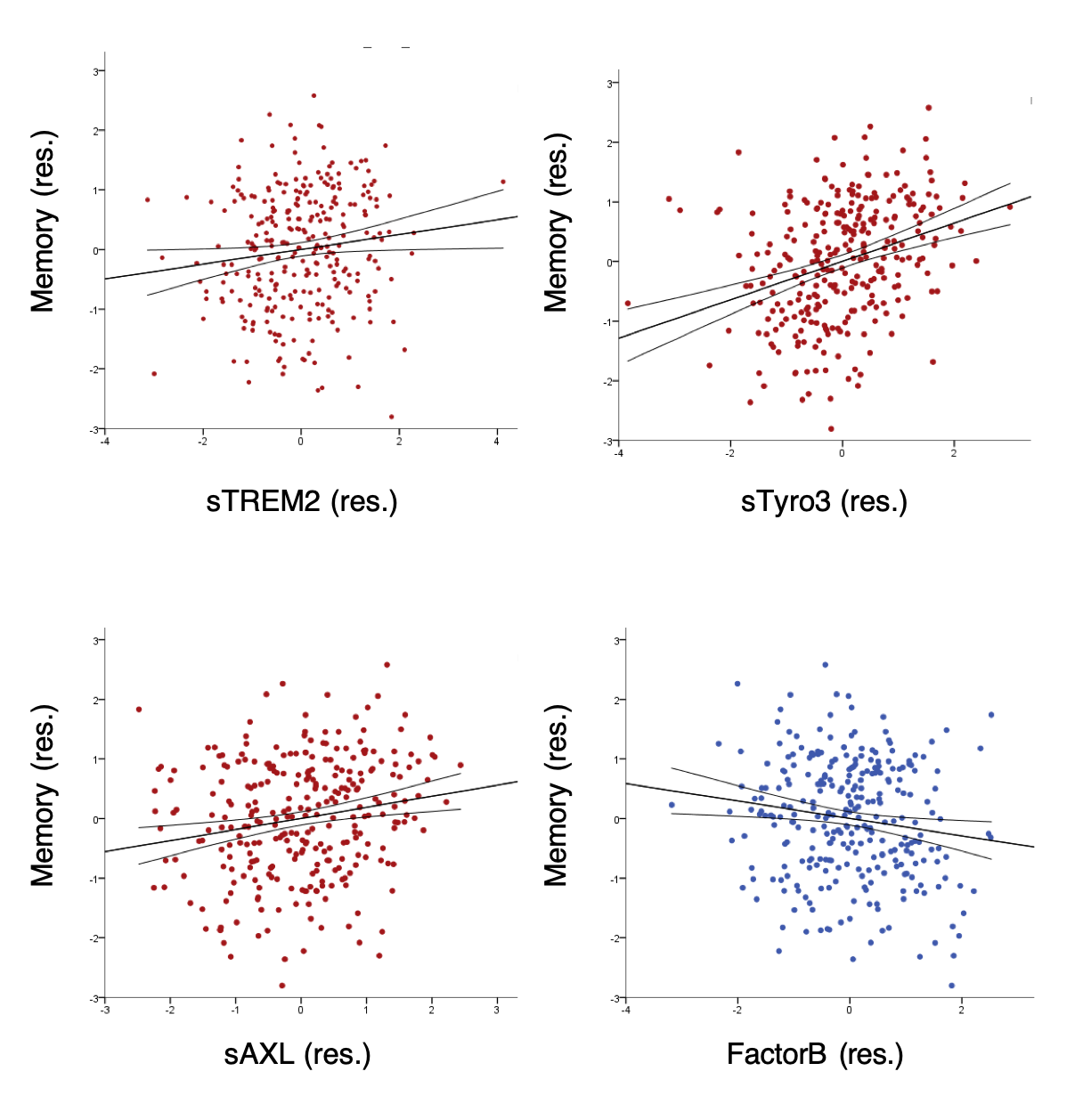


S**upplementary Figure 4. Associations of individual inflammation-related markers with memory.** Scatter plots for significant associations of individual markers (see Supplementary correlation table 3) loading on PC1 (sTREM2, sTyro3 and sAXL) or PC2 (Factor B) with memory performance at baseline in the DELCODE sample. Standardized residuals are shown after regressing out covariates (age, gender, p-tau181, Aβ42/40).

# Supplementary Tables

**Supplementary Table 1. Principal component analysis on inflammatory markers before rotation**

| CSF Marker | PC1 | PC2 | PC3 | PC4 |
| --- | --- | --- | --- | --- |
| Factor H | **.853** | .242 |  |  |
| C4 | **.804** |  |  |  |
| C1q | **.726** | -.223 |  |  |
| Factor B | **.694** | .446 |  | -.366 |
| MIF | **.658** | -.268 | -.411 | .268 |
| YKL40 | **.657** | -.359 |  |  |
| Il18 | **.633** |  | .267 | -.282 |
| IP10 | **.509** | .221 | -.357 | .307 |
| Tyro3 | .409 | **-.739** | .221 |  |
| AXL | .500 | **-.691** |  |  |
| TREM2 | .481 | **-.601** |  |  |
| CRP | .290 | **.499** |  | -.478 |
| Il6 | .383 | **.430** |  | .297 |
| C3 | .307 | .368 | **.665** | .490 |
| C3b | .559 | .332 | **.624** | .298 |
| MCP1 | .495 | .224 | **-.587** | .317 |

PCA components and their loadings before orthogonal rotation in the DELCODE sample. Final principal components after (varimax) rotation are shown in Supplementary Figure 2.

**Supplementary Table 2. Correlations of inflammatory PCs with demographics and AD biomarkers**

|  | Statistic | age | Education (years) | BMI | Aβ42/40 | p-tau181 | FCRP |
| --- | --- | --- | --- | --- | --- | --- | --- |
| PC1 | r /rho | **0.281** | 0.065 | **-0.218** | **-0.187** | **0.627** | -0.026 |
|  | P | **<0.001**** | 0.265 | **<0.001**** | **0.001**** | **<0.001**** | 0.685 |
|  | N | **295** | 295 | **294** | **295** | **295** | 237 |
| PC2 | r /rho | 0.038 | 0.012 | **0.226** | 0.075 | -0.054 | **0.145** |
|  | P | 0.516 | 0.842 | **<0.001**** | 0.199 | 0.357 | **0.026*** |
|  | N | 295 | 295 | **294** | 295 | 295 | **237** |
| PC3 | r /rho | **0.126** | -0.034 | 0.012 | -0.026 | *0.111* | **0.144** |
|  | P | **0.030*** | 0.562 | 0.839 | 0.651 | *0.057* | **0.027*** |
|  | N | **295** | 295 | 294 | 295 | *295* | **237** |
| PC4 | r /rho | 0.074 | 0.029 | **0.129** | -0.063 | 0.030 | 0.110 |
|  | P | 0.203 | 0.623 | **0.027*** | 0.277 | 0.608 | 0.090 |
|  | N | 295 | 295 | **294** | 295 | 295 | 237 |

Descriptive correlations of the principal components derived from the 16 inflammatory markers with demographics and biomarkers of AD pathology in the DELCODE sample. Shown are Pearson r-values for all variables except for the FCRP (Framingham cardiovascular risk profile score), where Spearman rho values are shown. *p<0.05 (uncorrected, also highlighted in bold), **p<0.05 (corrected for multiple comparisons). When controlling for p-tau, PC1 related to higher Aβ42/40 (r = .266, p<0.001) with no significant correlation for any other PC (all p>0.3).

**Supplementary Table 3. Partial correlations of individual inflammatory markers loading on PC1 and PC2 with brain measures and cognition**

|  | Marker | Stats | GM volume | WM volume | WMH volume | GM rate of change | Memory | PACC5 change |
| --- | --- | --- | --- | --- | --- | --- | --- | --- |
| PC1 | sTREM2 | r | 0.048 | *0.116* | *-0.114* | -0.118 | **0.137*** | 0.063 |
|  |  | P | 0.449 | *0.066* | *0.075* | 0.125 | **0.019** | 0.376 |
|  |  | DF | 251 | *251* | *242* | 167 | **289** | 196 |
|  | YKL-40 | r | -0.068 | -0.019 | -0.008 | -0.045 | -0.049 | 0.090 |
|  |  | P | 0.281 | 0.764 | 0.903 | 0.559 | 0.410 | 0.208 |
|  |  | DF | 251 | 251 | 242 | 167 | 289 | 196 |
|  | sAXL | r | **0.189**** | *0.108* | **-0.209**** | **-0.235**** | **0.179**** | **0.180*** |
|  |  | P | **0.003** | *0.087* | **0.001** | **0.002** | **0.002** | **0.011** |
|  |  | DF | **251** | *251* | **242** | **167** | **289** | **196** |
|  | sTyro3 | r | **0.306**** | **0.232**** | **-0.185**** | **-0.278**** | **0.321**** | **0.212**** |
|  |  | P | **<0.001** | **<0.001** | **0.004** | **<0.001** | **<0.001** | **0.003** |
|  |  | DF | **251** | **251** | **242** | **167** | **289** | **196** |
|  | C1q | r | 0.011 | -0.086 | 0.037 | -0.072 | -0.026 | 0.086 |
|  |  | P | 0.868 | 0.174 | 0.570 | 0.349 | 0.662 | 0.228 |
|  |  | DF | 251 | 251 | 242 | 167 | 289 | 196 |
| PC2 | C4 | r | -0.080 | -0.032 | 0.044 | *-0.148* | -0.096 | -0.048 |
|  |  | P | 0.202 | 0.614 | 0.491 | *0.054* | 0.103 | 0.499 |
|  |  | DF | 251 | 251 | 242 | *167* | 289 | 196 |
|  | CRP | r | **-0.174** | -0.103 | 0.022 | *-0.137* | **-0.123*** | *-0.131* |
|  |  | P | **0.006**** | 0.104 | 0.732 | *0.077* | **0.037** | *0.065* |
|  |  | DF | **251** | 251 | 242 | *167* | **289** | *196* |
|  | IL-18 | r | **-0.150*** | -0.088 | 0.028 | -0.076 | -0.027 | 0.120 |
|  |  | P | **0.017** | 0.165 | 0.663 | 0.326 | 0.645 | 0.091 |
|  |  | DF | **251** | 251 | 242 | 167 | 289 | 196 |
|  | Factor B | r | **-0.159*** | **-0.152*** | -0.015 | *-0.143* | **-0.157**** | -0.098 |
|  |  | P | **0.011** | **0.016** | 0.810 | *0.064* | **0.007** | 0.170 |
|  |  | DF | **251** | **251** | 242 | *167* | **289** | 196 |
|  | Factor H | r | *-0.109* | **-0.136*** | 0.018 | -0.103 | **-0.116*** | -0.082 |
|  |  | P | *0.085* | **0.030** | 0.784 | 0.183 | **0.048** | 0.254 |
|  |  | DF | *251* | **251** | 242 | 167 | **289** | 196 |

Supplementary partial correlations of individual inflammatory markers loading on PC1 and PC2 with brain measures and cognition covaried for age, gender, p-tau181, Aβ42/40 and ICV (for volume measures) in the DELCODE sample. DF=degrees of freedom. P values are uncorrected. Significant correlations at p<0.05* (uncorrected) are highlighted in bold and trend level associations in italic. **p<0.05 (corrected for multiple comparisons). Supplementary Figure 4 additionally shows scatter plots for significant marker association with memory.

**Supplementary Table 4. Partial correlations of domain-specific cognitive scores with inflammatory components PC1 and PC2**

|  |  | Episodic Memory | Language | Executive Function | Working Memory | Visual | Global Cognition | MMSE |
| --- | --- | --- | --- | --- | --- | --- | --- | --- |
| PC1 | r | **0.230**** | **0.200**** | **0.209*** | **0.120*** | **0.165**** | **0.202**** | **.173**** |
|  | P | **<0.001** | **0.001** | **<0.001** | **0.041** | **0.005** | **0.001** | **.003** |
|  | DF | **289** | **289** | **289** | **289** | **289** | **289** | **289** |
| PC2 | r | **-0.172**** | **-0.139**** | **-0.119*** | **-0.110*** | -0.048 | **-0.130*** | **-.127*** |
|  | P | **0.003** | **0.018** | **0.042** | **0.060** | 0.411 | **0.026** | **0.031** |
|  | DF | **289** | **289** | **289** | **289** | 289 | **289** | **289** |

Supplementary partial Pearson correlations of inflammatory components PC1 and PC2 with the different cognitive domain scores and MMSE covaried for age, gender, p-tau181, and Aβ42/40 in the DELCODE sample. DF=degrees of freedom. P values are uncorrected. Significant correlations at p<0.05* (uncorrected) are highlighted in bold and trend level associations in italic. **p<0.05 (corrected for multiple comparisons).

**Supplementary Table 5. Sample characteristics of F.ACE cohort**

| Feature | all | Cognitively unimpaired (CU) | Cognitively impaired (CI) |
| --- | --- | --- | --- |
| N | 184 | 56 | 128 |
| Diagnostic groups (N) | SCD+MCI | SCD | MCI |
| age (yrs.) | 65.5±6.9 | 65.5±6.2 | 65.5±7.3 |
| N female (%) | 103 (56) | 32(57.1) | 71(55.5) |
| BMI | 26.9±4.2 | 26.2±3.6 | 27.3±4.4 |
| N APOE ε4+ (%) | 36 (19.6) | 5 (8.9) | 31 (24.2) |
| Aβ42 | 1010.3±337.5 | 1061.8±295.4 | 987.8±353.1 |
| N Aβ42/40 (%) | 87 (47.3) | 15 (26.8) | 72 (56.2) |
| Aβ42/40 | 0.08±0.02 | 0.09±0.01 | 0.08±0.02 |
| p-tau181 (pg/ml) | 51.8±29.2 | 43.9±14.4 | 55.3±33.2 |
| N A-T- (%) | 116 (63) | 45 (80.4) | 71 (55.5) |
| N A+T- (%) | 21 (11.4) | 4 (7.1) | 17 (13.3) |
| N A-T+ (%) | 25 (13.6) | 4 (7.1) | 21(16.4) |
| N A+T+ (%) | 22 (12) | 3 (5.4) | 19 (14.8) |
| MMSE | 28.3±1.8 | 29.5±0.7 | 27.8±1.9 |
| Memory Z-score | 0.057±0.93 | 0.8±0.6 | -0.2±0.9 |

Unless otherwise stated variables denote mean ± standard deviation. Percentages are based on number of valid cases. MMSE = Mini-Mental State Examination; APOE ε4 = carriers of at least one apolipoprotein E ε4 allele. A memory Z-score was computed using a z-composite score of the Wechsler memory scale III immediate and delayed word list recall.

**Supplementary Table 6. Regression model for GM thickness predicted by inflammatory components in F.ACE**

| dependent | independent | F | P | Partial eta2 | B | SE | T |
| --- | --- | --- | --- | --- | --- | --- | --- |
| GM thickness | gender | 0.161 | 0.688 | 0.001 | -0.011 | 0.027 | -0.402 |
|  | age* | 12.879 | <0.001 | 0.069 | -0.007 | 0.002 | -3.589 |
|  | **PC1*** | **7.556** | **0.007** | **0.042** | **0.059** | **0.022** | **2.749** |
|  | **PC2*** | **4.017** | **0.047** | **0.023** | **-0.027** | **0.013** | **-2.004** |
|  | Aβ42 | 1.032 | 0.311 | 0.006 | 0.000 | 0.000 | -1.348 |
|  | p-tau* | 6.793 | 0.010 | 0.038 | -0.002 | 0.001 | -3.231 |
|  | assay | 0.873 | 0.351 | 0.005 | -0.094 | 0.101 | -0.934 |
|  | assay x Aβ42 | 0.280 | 0.597 | 0.002 | 0.000 | 0.000 | 0.529 |
|  | assay x P-tau | 0.113 | 0.737 | 0.001 | 0.000 | 0.001 | 0.337 |

Regression models tested whether inflammatory components PC1 and PC2 derived from a PCA predicted GM thickness in AD-vulnerable regions derived from FreesSurfer segmentation (including bilateral entorhinal, parahippocampal, fusiform, inferior temporal, temporal pole and posterior cingulate). Significant (*) effects of interest are highlighted in bold. Additional covariates of no interest in all models included age, gender, p-tau and Aβ42.

**Supplementary Table 7. Regression model for memory performance at baseline predicted by inflammatory components in F.ACE**

| dependent | independent | F | P | Partial eta2 | B | SE | T |
| --- | --- | --- | --- | --- | --- | --- | --- |
| MMSE | gender | 1.058 | 0.305 | 0.007 | -0.288 | 0.280 | -1.028 |
|  | Age | 0.815 | 0.368 | 0.005 | -0.019 | 0.021 | -0.903 |
|  | **PC1*** | **11.657** | **<0.001** | **0.069** | **0.766** | **0.224** | **3.414** |
|  | PC2 | 0.180 | 0.672 | 0.001 | -0.058 | 0.137 | -0.424 |
|  | Aβ42 | 0.403 | 0.527 | 0.003 | 0.001 | 0.001 | 1.072 |
|  | p-tau* | 16.380 | <0.001 | 0.094 | -0.026 | 0.007 | -4.028 |
|  | assay | 2.249 | 0.136 | 0.014 | 1.558 | 1.039 | 1.500 |
|  | assay x Aβ42 | 0.408 | 0.524 | 0.003 | -0.001 | 0.001 | -0.639 |
|  | assay x Ptau | 0.470 | 0.494 | 0.003 | -0.007 | 0.011 | -0.686 |
| Memory score | gender* | 7.626 | 0.006 | 0.046 | -0.382 | 0.138 | -2.762 |
|  | age* | 7.627 | 0.006 | 0.046 | -0.028 | 0.010 | -2.762 |
|  | **PC1*** | **7.182** | **0.008** | **0.043** | **0.297** | **0.111** | **2.680** |
|  | PC2 | 1.066 | 0.303 | 0.007 | -0.070 | 0.068 | -1.033 |
|  | Aβ42 | 0.533 | 0.467 | 0.003 | 0.000 | 0.000 | 0.888 |
|  | p-tau* | 12.136 | <0.001 | 0.071 | -0.013 | 0.003 | -4.101 |
|  | assay | 0.104 | 0.747 | 0.001 | 0.166 | 0.513 | 0.323 |
|  | assay x Aβ42 | 0.078 | 0.780 | <0.001 | 0.000 | 0.000 | -0.280 |
|  | assay x p-tau | 0.040 | 0.842 | <0.001 | 0.001 | 0.005 | 0.200 |

Regression models tested whether inflammatory components PC1 and PC2 predicted episodic memory or MMSE at baseline (N = 184). The memory score was computed using a z-composite score of the Wechsler memory scale III immediate and delayed word list recall. Significant (*) effects of interest are highlighted in bold.

**Supplementary Table 8. LME for cognitive change predicted by inflammatory markers**

| Model | dependent | independent | P | CI | Estimate |
| --- | --- | --- | --- | --- | --- |
| 1 | composite | age* | 0.026 | -0.29 – -0.02 | -0.16 |
|  |  | p-tau181* | 0.046 | -0.02 – -0.00 | -0.01 |
|  |  | time x age* | 0.004 | -0.11– -0.0.2 | -0.06 |
|  |  | **PC1*** | **0.011** | **0.06 – 0.43** | **0.25** |
|  |  | PC2 | 0.850 | -0.16-0.13 | -0.1 |
|  |  | time x PC1* | 0.017 | 0.01 – 0.13 | 0.07 |
|  |  | time x PC2 | 0.205 | -0.01-0.07 | 0.03 |
| 2 | composite | age* | 0.042 | -0.28 – -0.01 | -0.14 |
|  |  | time x age* | 0.021 | -0.10 – -0.01 | -0.05 |
|  |  | PC1 | 0.143 | -0.06 – 0.40 | 0.17 |
|  |  | PC2 | 0.860 | -0.16 - 0.13 | -0.1 |
|  |  | time x PC1 | 0.288 | -0.03 – 0.11 | 0.04 |
|  |  | time x PC2 | 0.328 | -0.02 – 0.06 | 0.02 |

For effects on longitudinal cognitive change, LMEs (linear mixed effects model) were run to predict PACC5 scores (N = 180, N observations = 468). Model 1 included as additional covariates age, gender, p-tau181, assay type, p-tau*assay interaction as well as interactions of each predictor with time. Model 2 further included Aβ42, and Aβ42 *assay interaction as well as its interactions with time. For simplicity, for covariates only significant effects (*) are shown. For our variables of interest PC1 and PC2 all results are shown (also if not significant). Significant effects of interest are highlighted in bold.

# References

1. Jack, C.R., Jr., et al., *Defining imaging biomarker cut points for brain aging and Alzheimer's disease.* Alzheimers Dement, 2017. **13**(3): p. 205-216.

2. Guo, T., et al., *Normalization of CSF pTau measurement by Abeta(40) improves its performance as a biomarker of Alzheimer's disease.* Alzheimers Res Ther, 2020. **12**(1): p. 97.

3. Karlsson, L.V., J.; Arvidsson, I.; Åström, K.; Janelidze, S.; Blennow, K.; Palmqvist, S.; Stomrud, E.; Mattsson-Carlgren, N.; Hansson, O., *Cerebrospinal fluid reference proteins increase accuracy and interpretability of biomarkers for brain diseases.* Preprint, 2023.

4. Boada, M., et al., *Design of a comprehensive Alzheimer's disease clinic and research center in Spain to meet critical patient and family needs.* Alzheimers Dement, 2014. **10**(3): p. 409-15.

5. Brosseron, F., et al., *Soluble TAM receptors sAXL and sTyro3 predict structural and functional protection in Alzheimer's disease.* Neuron, 2022. **110**(6): p. 1009-1022 e4.

6. Espinosa, A., et al., *A longitudinal follow-up of 550 mild cognitive impairment patients: evidence for large conversion to dementia rates and detection of major risk factors involved.* J Alzheimers Dis, 2013. **34**(3): p. 769-80.

7. Rodriguez-Gomez, O., et al., *FACEHBI: A Prospective Study of Risk Factors, Biomarkers and Cognition in a Cohort of Individuals with Subjective Cognitive Decline. Study Rationale and Research Protocols.* J Prev Alzheimers Dis, 2017. **4**(2): p. 100-108.
